# Age-Dependent Effects of Heavy Metals on the Hypothalamic–Pituitary–Testicular Axis-Related Hormones in Men

**DOI:** 10.3390/toxics14010055

**Published:** 2026-01-07

**Authors:** Yayuan Mei, Yongfu Yan, Shenglan Ke, Weihui Su, Zhangjia Luo, Xiaobao Chen, Hui Xu, Weitao Su, Ang Li

**Affiliations:** 1 Big Data Center, Beijing Children’s Hospital, Capital Medical University, National Center for Children’s Health, Beijing 100045, China; mei809000@163.com (Y.M.); xuhuiyunyun@126.com (H.X.); 2 Chinese Center for Disease Control and Prevention, Beijing 100050, China; yongfu_yan97@163.com; 3 Xianning Medical College, Hubei University of Science and Technology, Xianning 437100, China; 13317291995@163.com (S.K.); 19877805312@163.com (W.S.); 17788904124@163.com (Z.L.); 13227311643@163.com (X.C.); 4 Central Laboratory, The Second Affiliated Hospital of Fujian Medical University, Quanzhou 362000, China; suweitao@fjmu.edu.cn; 5 Hebei Key Laboratory of Environment and Human Health, Shijiazhuang 050017, China; 6 Department of Epidemiology and Statistics, School of Public Health, Hebei Medical University, Shijiazhuang 050017, China

**Keywords:** metal elements, sex hormones, joint effect, male, vitamin D

## Abstract

The effect of heavy metals on male hormonal regulation—particularly the hypothalamic–pituitary–testicular (HPT) axis—remains poorly characterized. We aim to investigate associations between heavy metal exposure and HPT axis-related hormones. We analyzed data, including male participants aged 3–80 years, from a nationally representative survey. Five metals and twelve sex hormones were measured. We used multivariate linear regression and restricted cubic splines to assess associations and dose–response relationships. Mixture effects were quantified using quantile-based g computation. The modifying effects of vitamin D and folate were examined. The underlying mechanisms were explored through a narrative review and integrative bioinformatics analysis. A total of 6547 males were included. Metal exposure was predominantly associated with hormonal perturbations in adolescents and older adults. Specifically, metal mixture was associated with hormones in adolescent males [effect range: −5.10% (95% CI: −9.24, −0.76) to 18.12% (95% CI: 9.80, 27.07)] and older males [effect range: 3.17% (95% CI: 0.07, 6.37) to 10.94% (95% CI: 4.82, 17.43)]. Effect modifications were observed for vitamin D in children and adolescents, and for folate across all age groups. The *PI3K-Akt* signaling pathway was identified as a potential mechanism. Our findings provide novel insights into the association and potential pathway between heavy metals and male hormonal disturbance.

## 1. Introduction

Metal ions universally exist in the human environment (e.g., air, soil, water, dust, industrial products, or food) and participate in multifaceted physiological and pathological processes related to human health. Some heavy metals, such as selenium (Se) and manganese (Mn), are essential, in trace amounts, for metabolism, growth, reproduction, and immune response, while they have negative effects when they are deficient or excessive [1,2]. In contrast, non-essential elements, such as cadmium (Cd), lead (Pb), and mercury (Hg), may cause multi-organ toxicity, even at low exposure levels [3,4,5]. Cd is an estrogen disruptor that induces thyrotoxicity by promoting thyroid follicular cell pyroptosis [6]. Pb is an endocrine disruptor ubiquitous in industrial products and typically causes irreversible health damage [6]. Hg, a unique metal that exists primarily in nature as compounds, has demonstrated broad toxicity in humans, including neurotoxicity, reproductive toxicity, histone modification, gene damage, and epigenetic methylation [6]. Like the effect of endocrine-disrupting chemicals (EDCs), metal exposure potentially affects human hormone regulation [7,8].

Hormones are signaling molecules secreted by glands or endocrine tissues, which multicellular organisms use to regulate, coordinate, and integrate cellular and tissue physiological functions [9,10]. Both hormone production and action are controlled by complex regulatory mechanisms, with significant age-related variations in males. Under the hypothalamus–pituitary–testicular (HPT) axis, the gonadotropins follicle-stimulating hormone (FSH) and luteinizing hormone (LH) are regulated by pulsatile gonadotropin-releasing hormone (GnRH) secretion [11,12]. In males, sustained secretion of LH maintains the production of Leydig cell testosterone (TST), while FSH acts on Sertoli cells to regulate seminiferous tubule function and spermatogenesis [11]. Meanwhile, TST and estradiol (EST) exert negative feedback to inhibit the secretion of GnRH and LH, with a similar pattern on FSH by inhibin B regulation [11]. Dysfunction of the HPT axis impairs male reproductive function, characterized by reduced sperm concentration, total sperm count, motility, and viability, along with increased abnormal sperm morphology and DNA fragmentation, ultimately contributing to male infertility [13,14]. Under the hypothalamic–pituitary–adrenal (HPA) axis, dehydroepiandrosterone sulfate (DHE) and cortisol are released through adrenocorticotropic hormone (ACTH) stimulation of adrenal steroidogenesis, and 17α-hydroxyprogesterone (17H) is produced as an intermediate within this pathway [10,15]. The cortisol and DHE produce negative feedback to inhibit further activation of the axis to reduce the corticotropin-releasing hormone (CRH) and ACTH secretion [10,16]. When ACTH is secreted ectopic, the risk of Cushing’s syndrome, obstructive sleep apnea, depression, and *abnormal inflammatory* responses significantly increases [17]. 17H is a cortisol precursor with a deficiency in males that can develop into precocious puberty [18].

Increasing evidence indicates that heavy metals have an unfavorable effect on the regulation of sex hormones [7,19,20,21]. However, existing evidence revealed that few studies have focused on the association between individual heavy metal exposure and male reproductive hormones across different ages, let alone explored the effect of exposure to metal mixtures. In addition, current studies have not adequately demonstrated the dose–response relationships between heavy metal exposure and male hormones. A study using nationally representative U.S. participants aged ≥ 18 years explored the dose-response relationships between metals and male sex hormones [21], but they did not stratify by the different age groups. Finally, although some hypotheses have been proposed, the precise underlying mechanisms remain incompletely elucidated.

Vitamin D and folate are crucial nutrients, potentially exhibiting protective effects by reducing heavy metal absorption and toxicity, while also promoting detoxification and excretion [22,23,24]. Specifically, adequate vitamin D can induce the mRNA expression of metallothionein, which participates in the transport, storage, and detoxification of essential and non-essential metal ions [22]. One-carbon metabolism requires folate and is involved in S-adenosylmethionine production, which is significantly associated with DNA methylation (a potential mechanism linking metals and hormones) [24]. Previous studies also suggested that vitamin D and folate can modify the association between environmental factors (e.g., aldehydes) and sex hormones [25]. Therefore, the modifying role of these two factors should be explored for the hormonal effect of metals.

Therefore, the purpose of this study was to examine the interconnection of individual and combined heavy metal (Pb, Cd, Hg, Se, and Mn) exposure with sex hormones [17H, androstenedione (AND), DHE, estrone (ESO), estrone sulfate (ES1), EST, progesterone (PG4), TST, (AMH), LH, FSH, and SHBG] in males, while identifying the modifying roles of vitamin D and folate. We further explored the underlying mechanisms linking metal exposure and male hormonal disturbance based on a narrative review and bioinformatic analysis.

## 2. Materials and Methods

### 2.1. Study Design and Population

The National Health and Nutrition Examination Survey (NHANES) is a nationally representative, cross-sectional study continuously conducted since 1999 by the National Center for Health Statistics (NCHS) of the Centers for Disease Control and Prevention. It includes health exams, laboratory tests, and dietary interviews for participants of all ages to assess the health and nutritional status of the United States’ population. The survey protocol was approved by the Research Ethics Review Board of the NCHS. All participants provided written informed consent prior to survey commencement. This study included data from cycles 2017–2023 of NHANES, with a total of 27,493 participants aged 3 to 80 years. Participants who had missing values for metals (*n* = 14), hormones (*n* = 4139), or demographic information (*n* = 9710), and females (*n* = 7083), were excluded. Finally, 6547 males were eligible for subsequent analysis (see Figure S1).

### 2.2. Assessment of Metal Exposure

In the 2017–2023 cycle, only blood concentrations of Pb, Cd, Hg, Se, and Mn are available. Blood samples were collected by a phlebotomist and put into vials for storage at a mobile examination center. Anti-coagulant EDTA (ethylenediaminetetraacetic acid) was added to the blood collection tube before collection [26]. Afterwards, the vials were then frozen at −30 °C before being transported to the laboratories at the National Center for Environmental Health for analysis. The target metals (Pb, Cd, Hg, Se, and Mn) were assayed by inductively coupled plasma mass spectrometry (ICP-MS) [26]. The internal standards, rhodium, iridium, and tellurium, were at a constant concentration in all blanks, calibrators, and samples. Monitoring the instrument signal ratio of a metal to its internal standard allows correction for instrument noise and drift, and sample-to-sample matrix differences [26]. The lower limits of detection (LOD) were 0.065 µg/L for Cd, 0.049 µg/dL for Pb, 0.52 µg/L for Mn, 0.17 µg/L for Hg, and 9.90 µg/L for Se. The proportions above the LOD were 99.98% for Pb, 86.76% for Cd, 71.06% for Hg, 100% for Se, and 100% for Mn. Metal concentrations lower than LOD were substituted with the value of LOD/√2.

### 2.3. Measurement of Hormones, Vitamin D, and Folate

Serum concentrations of steroid hormones [17H, AND, DHE, ESO, ES1, EST, PG4, and TST] were quantified using four main steps based on isotope-dilution liquid chromatography–tandem mass spectrometry (ID LC-MS/MS) [26]. Levels of AMH, LH, FSH, and SHBG were measured through two incubation steps and a chemiluminescent measurement via photomultiplier tube [26]. The LOD for the analyzed hormones was as follows: 17H: 0.41 ng/dL; AND: 0.82 ng/dL; AMH: 0.03 ng/mL; DHE: 0.22 ug/dL; EST: 1.72 pg/mL; ESO: 0.13 ng/dL; ES1: 2.04 pg/mL; FSH: 0.300 mIU/mL; LH: 0.100 mIU/mL; PG4: 0.86 ng/dL; SHBG: 0.350 nmol/L; and TST: 0.57 ng/dL.

To investigate the modifying effects of folate and vitamin D, their serum levels were measured. Folate was measured via isotope-dilution liquid chromatography coupled to tandem mass spectrometry (LC-MS/MS), and vitamin D was measured by high-performance liquid chromatography–tandem mass spectrometry (HPLC-MS/MS) [26]. The detection of these substances has undergone rigorous method validation and adhered to stringent quality control standards and strict operating procedures.

### 2.4. Assessments of Covariates

Baseline characteristics were included as covariates to control for confounding bias: age and body mass index (BMI) were treated as continuous variables; education level was classified into three groups (high school or below, some college or associate’s degree level, and college graduate or above); race/ethnicity was categorized as four groups (Hispanic, non-Hispanic White, non-Hispanic Black, and other races); marital status was categorized as married/with partner or separated/never married; and examination time was divided into morning or afternoon/evening. Participants who reported never smoking or smoked ≤100 cigarettes in their lifetime were considered as never smokers, those who smoked >100 cigarettes in their lifetime and now smoked cigarettes every day or sometimes were considered as current smokers, and those who smoked >100 cigarettes and had quit smoking were considered as former smokers. As for drinking habits, a never drinker was defined as never having drunk at least 12 alcoholic drinks in life, a former drinker was defined as having drunk at least 12 alcoholic drinks in life and quit drinking in the past year, and a current drinker was defined as having drunk at least 12 alcoholic drinks in life and still drank alcohol in the past year. Poverty was assessed by the poverty income ratio (PIR) served as a continuous variable.

### 2.5. Statistical Analysis

Continuous variables were summarized as medians with interquartile ranges (IQRs; 25th–75th percentiles) due to non-normal distributions, whereas categorical variables were expressed as frequencies and percentages. Univariate analyses were performed for continuous variables with the Wilcoxon rank sum test and categorical variables with the Chi-square test. The metals and hormones assay values were ln-transformed to normalize their distributions. Spearman’s correlation was used to evaluate the correlation coefficients among blood heavy metals and serum hormones.

To address the considerable age-related heterogeneity in hormone levels, all analyses were conducted stratified by age groups: children (3–11 years), adolescents (12–19 years), young adults (20–49 years), and older adults (50–80 years) [27]. Associations between single-metal exposures and hormone levels were assessed using three statistical approaches. Firstly, weighted multivariate linear regression models were applied based on the complex sampling design of NHANES, adjusting for baseline information (age, BMI, education level, race, marital status, examination time, smoking status, drinking status, and PIR). The adjusted regression coefficients for each metal were transformed as $\left(e^{ln2\times \beta }-1\right)\times 100\%$ and the 95% confidence intervals (CI) as $\left(e^{ln2\times (\beta \pm 1.96\times Se)}-1\right)\times 100\%$ to represent the percent changes in hormone levels per doubling of metal concentrations [28]. Secondly, the linear trend was assessed by grouping the blood metal concentrations into tertiles (as an ordinal variable), with the lowest tertile serving as the reference to examine the effect of each higher tertile. Finally, a restricted cubic spline (RCS) was used to explore potential exposure–response relationships with four knots (at the 5th, 35th, 65th, and 95th percentiles), while the reference was set at the 25th percentile metal value. To obtain robust findings, a relationship was only considered established if at least two consistent results were obtained.

The mixture effect of metal exposure on hormones was assessed by quantile g-computation, an approach that combines the inferential simplicity of WQS regression with the flexibility of g-computation, to accurately and precisely estimate the effects of mixtures [29]. We further investigated the modifying effects of folate and vitamin D using the 75th percentiles as cutoff points for stratified analysis to identify the complex interactions with heavy metal exposure on hormone levels. The statistical differences between high (≥75th percentile) and low levels (<75th percentile) were tested using ${(\beta }_{1}-\beta_{2})\pm 1.96\times \sqrt{{\left({Se}_{1}\right)}^{2}+{{(Se}_{2})}^{2}}$.

Sensitivity analyses were conducted by redefining the age groups for young adults and older adults at the cutoff points of 40 and 60 years, respectively, to assess the robustness of single-metal exposure results. Receiver operating characteristic (ROC) curves were employed to explore the predictive performance of blood hormones for heavy metal exposure. For each heavy metal significantly associated with hormone levels, we established cutoff values based on the 75th percentile of their concentration distributions [30].

All statistical analyses were performed using R software (version 4.4.1). Multiple testing was corrected using the false discovery rate (FDR), with statistical significance defined at FDR < 0.1. All statistical tests were two-sided, and *p* < 0.05 we considered statistically significant.

### 2.6. Bioinformatic Analysis

To better understand the findings, we explored the biological pathways through which heavy metals disrupt HPT axis-related hormones via a literature review and bioinformatic analysis.

Bioinformatics analysis utilized multiple database networks. Specifically, we retrieved genes associated with five metals (Cd, Pb, Mn, Hg, and Se) from the Comparative Toxicogenomics Database (CTD). Genes occurring in at least two of these metals were identified as common target genes. Subsequently, we extracted genes related to the HPT axis from both the GeneCards and Online Mendelian Inheritance in Man (OMIM) databases and took their union as the gene set associated with the HPT axis. Finally, we intersected the common metal target genes with the HPT axis-associated gene set to derive the ultimate gene set implicated in metal-induced HPT axis disruption. This set was then used for subsequent bioinformatics analyses. We used the STRING database to construct a protein–protein interaction (PPI) network for the gene set. This network was visualized using Cytoscape software (version 3.10.3), and hub genes involved in metal-induced hormone disruption were identified based on degree centrality. Additionally, Gene Ontology (GO) and Kyoto Encyclopedia of Genes and Genomes (KEGG) analyses were performed to investigate the phenotypically most relevant gene functions and potential pathways, respectively. We have provided a flow chart for this part (Figure S2).

## 3. Results

### 3.1. Baseline Characteristics

This study included 6547 males with a median age of 42 (IQR: 18–62) years old, stratified into four age groups: 824 (3–11 years old), 1044 (12–19 years old), 1924 (20–49 years old), and 2755 (50–80 years old) (Table 1). Of the participants, the median BMI was 26.8 (IQR: 22.6–31.4) kg/m^2^, 38.58% were educated at a high school level or below, 45.94% were non-Hispanic white, 62.77% marital status were married or with a partner, 50.39% were examined in the afternoon or evening, 50.12% were never smokers, 75.59% reported former drinking, and the median PIR was 2.5 (IQR: 1.3–4.6). The difference across age groups was significant in education level (*p* = 0.001), and in age, BMI, race, marital status, examined time, smoking habit, drinking habit, PIR, and metal exposure levels (all *p* < 0.0001). Correlation analysis showed that only SHBG was uncorrelated with PG4 and 17H, TST was uncorrelated with AMH, AMH was uncorrelated with Se, and DHE was uncorrelated with Mn, while other relationships were significant between hormones and with metals (Figure S3).

### 3.2. Distribution of Hormones Across Age Groups

Comparing the hormone levels across age groups, ESO, FSH, and LH show constant elevation with age (Figure S4). While EST and ES1 show an increasing trend for the whole age span, they maintain stability from age groups 20–49 to 50–80. TST, 17H, AND, PG4, and DHE increased levels from age groups 3–11 to 12–19. Except for AND, which increased dramatically from 12–19 to 20–49, others did not change substantially, and then all decreased from 20–49 to 50–80. SHBG showed a decreasing trend from age group 3–11 to 12–19, and did not significantly change from 12–19 to 20–49, whereas it increased from 20–49 to 50–80. Only AMH exhibits a constant downward trend with age.

### 3.3. Association Between Individual Heavy Metals and Hormones

**Figure 1:**
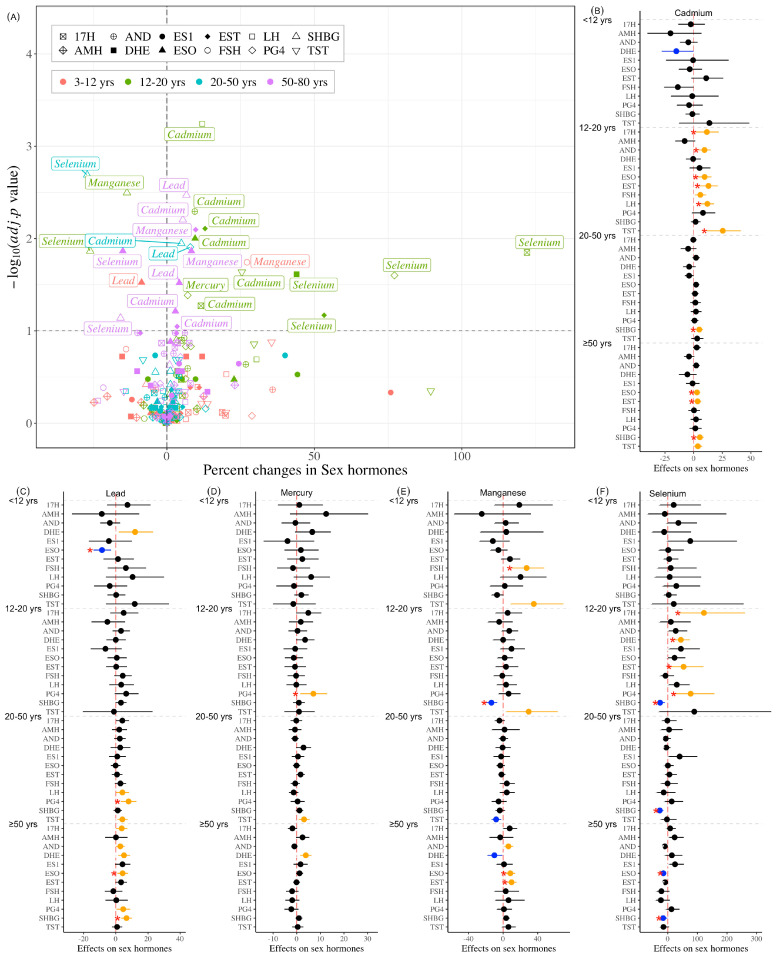
Association of individual metal exposure with male hormone profiles. Abbreviations: TST, testosterone; EST, estradiol; SHBG, sex hormone-binding globulin; 17H, 17α-hydroxyprogesterone; AND, androstenedione; AMH, anti-Müllerian hormone; ESO, estrone; ES1, estrone sulfate; FSH, follicle-stimulating hormone; LH, luteinizing hormone; PG4, progesterone; and DHE, dehydroepiandrosterone sulfate. Panel (**A**) is a volcano plot showing significant results after multiple corrections; panels (**B**–**F**) are error bar charts showing individual effects. Note: yellow bar indicates the positive association; blue bar indicates the negative association; black bar indicates non-significant associations; asterisk indicates the significant associations after multiple corrections.

The effects of single heavy metal exposure on hormones within different age groups are shown in Figure 1. For children, doubling of Pb exposure was associated with an 8.59% decrease in ESO (95% CI: −13.92–−2.94), while each doubling of Mn exposure was associated with a 27.16% increase in FSH (95% CI: 9.75–47.34). In adolescents, each fold increase in Cd exposure was associated with elevated levels of TST (25.41%; 95% CI: 11.38–41.21), EST (12.94%; 95% CI: 5.38–21.06), 17H (11.54%; 95% CI: 2.07–21.89), AND (9.46%; 95% CI: 4.19–14.98), ESO (9.54%; 95% CI: 3.85–15.55), and LH (11.98%; 95% CI: 6.43–17.83), while equivalent Se exposure increases showed positive associations with EST (53.32%; 95% CI: 6.69–120.34), 17H (122.11%; 95% CI: 36.62–261.11), PG4 (77.16%; 95% CI: 21.94–157.39), and DHE (43.98%; 95% CI: 18.74–74.59) but decreased SHBG (−26.02%; 95% CI: −39.38–−9.72), with Mn elevation demonstrating a negative association with SHBG (−13.55%; 95% CI: −19.87–−6.73) and Hg showing positive dose–response relationships with PG4 (7.05%; 95%CI: 1.53–12.86). In young adults, SHBG levels showed a positive association with Cd exposure (4.89%; 95% CI: 1.77–8.11) but a negative association with Se exposure (−27.05%; 95% CI: −37.42–−14.95), while PG4 was positively associated with Pb exposure (7.92%; 95% CI: 3.20–12.87). In older adults, Cd exposure showed positive associations with EST (3.46%; 95% CI: 0.38–6.62), SHBG (5.41%; 95% CI: 2.19–8.72), and ESO (2.89%; 95% CI: 0.14–5.72). Similarly, Mn exposure was linked to increased EST (9.75%; 95% CI: 4.19–15.61) and ESO (8.30%; 95% CI: 2.94–13.93), while Pb exposure correlated with higher SHBG (6.62%; 95% CI: 3.19–10.17) and ESO (4.21%; 95% CI: 0.91–7.61). In contrast, Se exposure exhibited inverse associations with SHBG (−15.72%; 95% CI: −28.06–−1.27) and ESO (−14.95%; 95% CI: −23.42–−5.53) (Figure 1).

**Figure 2:**
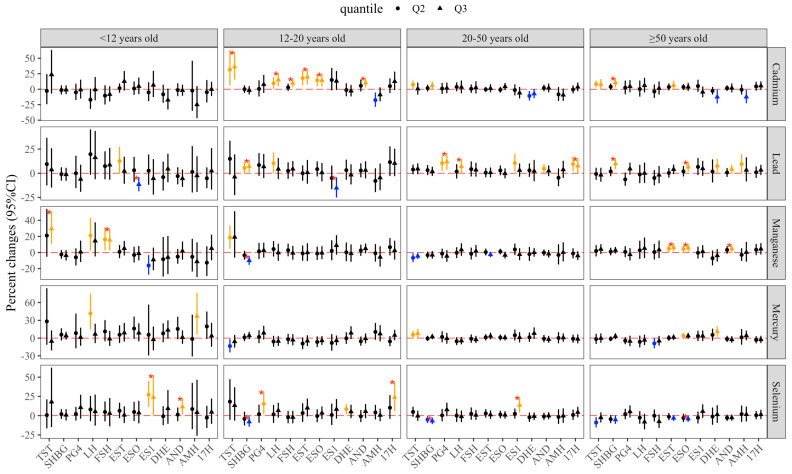
Association between different quantiles of metal exposure with hormones in different age groups. Abbreviations: TST, testosterone; EST, estradiol; SHBG, sex hormone-binding globulin; 17H, 17α-hydroxyprogesterone; AND, androstenedione; AMH, anti-Müllerian hormone; ESO, estrone; ES1, estrone sulfate; FSH, follicle-stimulating hormone; LH, luteinizing hormone; PG4, progesterone; and DHE, dehydroepiandrosterone sulfate. Note: yellow bar indicates significantly higher than the reference; blue bar indicates significantly lower than the reference; black bar indicates non-significant associations; asterisk indicates the P-for-trend was significant.

Figure 2 presents the linear trend test using the tertiles of metals as ordinal variables, and the individual tertile effects were also revealed by comparing with the lowest metal tertile. Figures S5–S8 demonstrate the nonlinear relationships between heavy metal exposure and hormone levels across age groups from the RCS model.

The robust results for individual metal exposure effects summarized from the previous three analyses are presented in Table 2. In children, Pb was negatively associated with ESO, and Mn was positively associated with FSH. In adolescents, Cd exposure was associated with increased levels of TST, EST, AND, ESO, and LH, and Se increase was associated with higher PG4 and DHE, but lower SHBG, and showed a nonlinear correlation with EST, with Mn negatively associated with SHBG and Hg positively associated with PG4. Moreover, Pb exhibited a nonlinear association with ES1, showing an initial increase, followed by a decrease and subsequent rebound. In young adults, Se exposure was negatively associated with SHBG and positively associated with ES1, and Pb was positively associated with LH and PG4. In parallel, Hg was positively associated with TST, and Cd was positively associated with SHBG. In older adults, Cd was positively associated with TST, EST, and ESO, and nonlinearly associated with SHBG; Mn was positively associated with EST, AND, and ESO, and Pb was nonlinearly associated with SHBG and positively associated with ESO. Similarly, Se was nonlinearly associated with SHBG but negatively associated with ESO. Hg showed a U-shaped exposure–response curve with FSH and was positively associated with DHE. In addition, the significant results among the three models in each age group are shown in Tables S1–S4.

### 3.4. Mixture Effects of Metal Exposure

**Figure 3:**
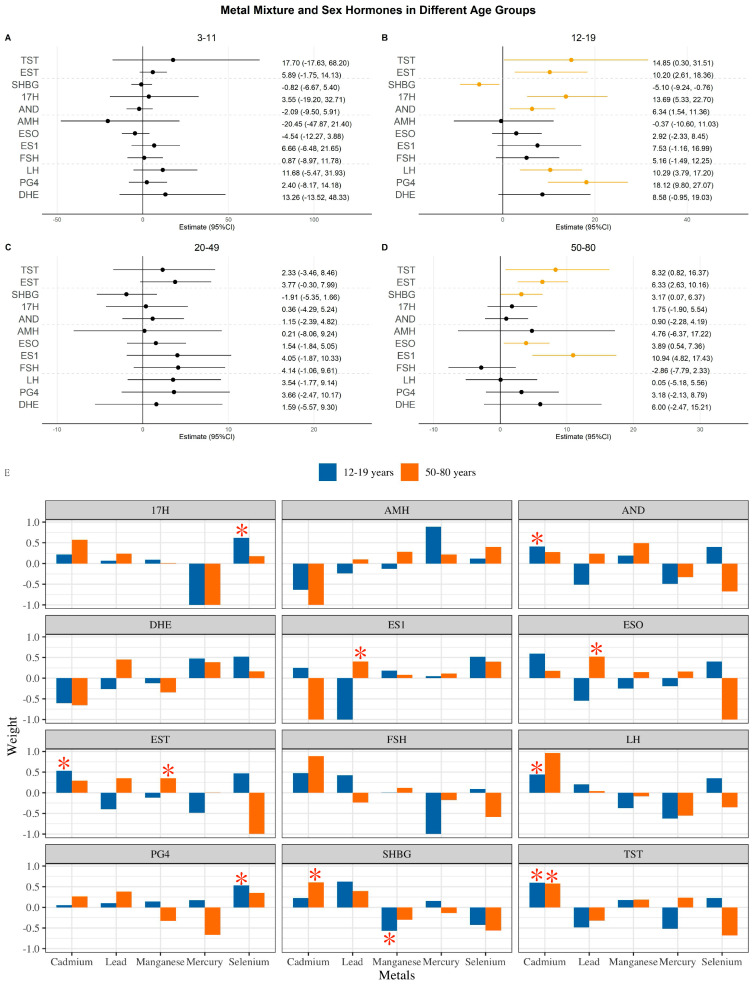
Association between metal mixture and hormones in different age groups. (**A**–**D**) Joint effect of metal mixture in different age groups; (**E**) weights for each metal in the joint effect model among adolescents and older adults. Note: asterisk indicates the most important metal in the mixture among each subgroup. Abbreviations: TST, testosterone; EST, estradiol; SHBG, sex hormone-binding globulin; 17H, 17α-hydroxyprogesterone; AND, androstenedione; AMH, anti-Müllerian hormone; ESO, estrone; ES1, estrone sulfate; FSH, follicle-stimulating hormone; LH, luteinizing hormone; PG4, progesterone; and DHE, dehydroepiandrosterone sulfate.

Using quantile g-computation to examine the effects of mixed metal exposure on hormones (Figure 3, Panel A–D), the effect was positively associated with TST in both 12–19 (β = 14.85; 95% CI: 0.30–31.51) and 50–80 (β = 8.32; 95% CI: 0.82–16.37) age groups, and also positively associated with EST in these age groups (12–19: β = 10.20; 95% CI: 2.61–18.36; 50–80: β = 6.33; 95% CI: 2.63–10.16). Moreover, mixed metal effects were positive in the age group 12–19 with 17H (β = 13.69; 95% CI: 5.33–22.7), AND (β = 6.34; 95% CI: 1.54–11.36), LH (β = 10.29; 95% CI: 3.79–17.2), and PG4 (β = 18.12; 95% CI: 9.80–27.07), and in the age group 50–80 with SHBG (β = 3.17; 95% CI: 0.07–6.37), ESO (β = 3.89; 95% CI: 0.54–7.36), and ES1 (β = 10.94; 95% CI: 4.82–17.43). We found that Cd accounted for most of the significant associations for joint exposure (Figure 3, Panel E).

### 3.5. Modifying Effects

**Figure 4:**
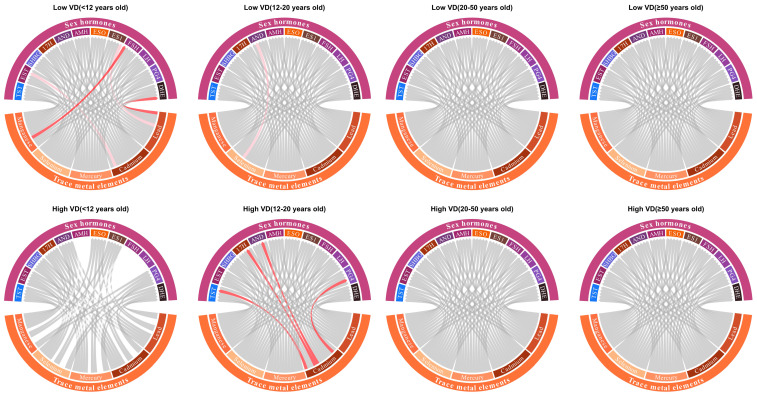
Effect modification of vitamin D in the association between metals and hormones. Abbreviations: TST, testosterone; EST, estradiol; SHBG, sex hormone-binding globulin; 17H, 17α-hydroxyprogesterone; AND, androstenedione; AMH, anti-Müllerian hormone; ESO, estrone; ES1, estrone sulfate; FSH, follicle-stimulating hormone; LH, luteinizing hormone; PG4, progesterone; and DHE, dehydroepiandrosterone sulfate. Note: The red line indicates a significant positive association between the corresponding metal-hormone pair, and a significant difference in effect estimates when compared to the same pair in the counterpart group. The pink line, by contrast, denotes a significant positive association for the pair but no significant difference in effect estimates relative to the same pair in the counterpart group.

Stratified analyses assessed the modifying effect of vitamin D and folate. As shown in Figure 4, the metal effect was mainly significant in age group 3–11 with lower blood vitamin D level, and significant in age group 12–19 with higher blood vitamin D level, but non-significant in the remaining age groups with both vitamin D levels. In participants with lower folate levels, the effects of metal exposure were primarily significant in age groups 12–19, 20–49, and 50–80 (Figure S9). However, the effects of metal exposure were rarely observed in participants with higher folate levels.

### 3.6. Biological Pathway of Metals on HPT Axis-Related Hormones

**Figure 5:**
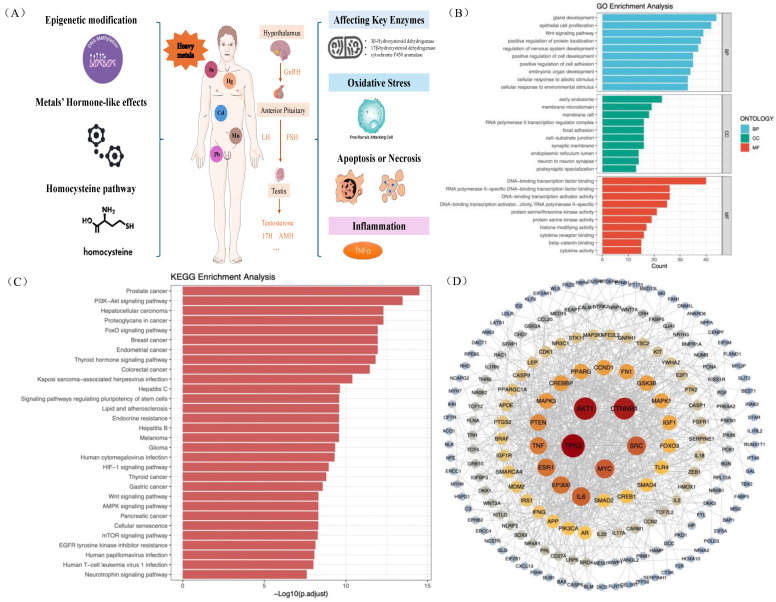
Potential pathways summary and bioinformatic analysis. (**A**) Potential pathways summary from a narrative review. (**B**) Top 30 GO terms of the target genes in the GO enrichment analysis. (**C**) Top 30 pathways of the target genes in the KEGG enrichment analysis. (**D**) The PPI network of potential targets for metals exposure caused sex hormone disturbance.

Several potential mechanisms were identified from a narrative literature review, including DNA methylation, oxidative stress, inflammation, metals’ hormone-like effects, affecting key enzymes, apoptosis and necrosis, and homocysteine pathways (Figure 5, Panel A).

Functional enrichment analysis was performed to explore underlying functions and enriched pathways (Figure 5, Panel B). We identified 2672 GO entries, including 2505 biological process (BP) terms, 63 cellular component (CC) terms, and 104 molecular function (MF) terms. Notably, gland development, cell development, Wnt signaling pathways, and cellular response to abiotic stimuli were implicated in metal-induced HPT axis disturbance.

KEGG enrichment analysis revealed 285 potential pathways (Figure 5, Panel C). The top 30 KEGG pathways indicated that metal exposure contributes to hormonal dysfunction through multiple mechanisms, including the PI3K-Akt signaling pathway, FoxO signaling pathway, Thyroid hormone signaling pathway, and Wnt signaling pathway. PPI network analysis identified 246 targets with intricate interactions, suggesting their potential role in metal-induced hormonal disruption (Figure 4, Panel D). The top three hub targets were TP53, AKT1, and CTNNB1.

### 3.7. Sensitivity Analyses

In the sensitivity analyses, the results for 40- and 60-year-old cutoff points were in overall agreement with the results of the 50-year-old cutoff point (Figure S10). For example, the associations between Cd and SHBG, and between Pb and PG4, were consistently positive, while Se and SHBG were negative in 20–39, 20–49, and 20–59 age groups. Likewise, the associations between Cd and SHBG, and Pb and PG4, were consistently positive in the 40–80, 50–80, and 60–80 age groups.

The predictive ability of hormones on associated metal is illustrated in Figure S11. Among children, there was no hormone AUC value higher than 0.7, whereas in young adults, SHBG has a good ability to predict Cd (AUC = 0.79), and LH was proficient in predicting Pb (AUC = 0.73). In adolescents, only DHE (AUC = 0.73) has great predictive ability in discriminating Se exposure. In older adults, SHBG showed better performance in predicting Cd (AUC = 0.75) and Pb (AUC = 0.72), while EST performed better in predicting Mn (AUC = 0.73).

## 4. Discussion

In this nationally representative cross-sectional study, the effects of individual and combined heavy metals on male hormones were primarily observed in the 12–19- and 50–80-year-old age groups. Additionally, the modifying effects of vitamin D on the association between heavy metal exposure and hormones were evident in the 3–11 and 12–19 age groups, whereas those of folate were observed across almost all age groups. The *PI3K-Akt* signaling pathway potentially links metal exposure to male hormone disturbance. Our findings highlight that heavy metal exposure in adolescents and older males deserves more attention.

In recent decades, researchers have documented significant reductions in human semen count and quality [31], with adverse effects on male fertility manifesting as both internal genetic expressions and external environmental disruptions [14]. Heavy metals are ubiquitous environmental contaminants that can cause adverse impacts on male fertility by disrupting the secretion of related hormones [32]. Similar to our findings, a study found a non-significant association between Se and TST in Chinese males [33]. Meeker et al. found that exposure to Se, Mn, Pb, Cd, and Hg was unassociated with FSH in a cross-sectional study [19]. Similarly, we did not observe any significant associations between metal mixture and FSH in any age subgroups. Chai et al. found that metal mixture exposure suppressed the levels of TST, FSH, LH, and PG4 in male college students [34]. Another study among male workers in a magnetic material factory revealed a positive association between Mn and LH, but a negative association between Mn and TST [35]. This is inconsistent with our findings regarding results for Mn. These discrepancies may come from variations in exposure levels across studies, as well as differences in population age, occupational factors, and analytical methods. Our study provided a comprehensive assessment of the associations between metal exposure and male hormones using multiple statistical models and a broad spectrum of hormones.

During childhood, the HPT axis in prepubertal males remains quiescent following its transient postnatal activation until the onset of puberty [11,36], thereby keeping sex hormones at low levels. Meanwhile, the HPA axis exhibits relative stability during this period, as it experiences lower stress and fewer interactions with sex hormones [16]. This stability may explain why the hormone levels in children, as shown in our results, are less susceptible to heavy metal exposure. Vitamin D is an essential nutrient and is crucial for children’s bone health [37,38], with an association found in pregnancy for lower maternal Cd, Mn, and Pb [39]. We found that vitamin D modified the adverse effects of Mn, Pb, and Cd on hormones in prepubertal males. In contrast, folate participates in a series of physiological processes, including biosynthesis, amino acid homeostasis, epigenetic maintenance, and redox defense [40]. A study using the NHANES data found that Cd, Pb, and Hg were significantly associated with red blood cell folate [41]. Further refinement with a well-designed study is warranted to validate our findings.

During adolescence, males experience HPT axis reactivation, the reproductive system develops into maturity [11], the interaction between the HPA and HPT axes increases activation [42], and physical growth occurs rapidly. This is the most vulnerable period affected by heavy metal exposure [43]. We found that adolescents’ TST, EST, 17H, AND, LH, and PG4 were disrupted by metal mixture exposure, and significant single-metal effects were observed for Cd, Se, Pb, Mn, and Hg. Vitamin D exhibited a marked modifying effect, with its high levels strengthening the associations between hormones and Cd. While studies showed negative correlations between Cd and vitamin D [23,44], the vitamin D during male puberty of the bone growth spurt promoted calcium absorption to a great extent, and we inferred that this procedure is partly replaced by Cd absorption as an “ionic mimicry” [45]. In addition, folate levels in pubertal males demonstrated a significant modifying effect in mitigating the adverse impacts of metal exposure. Current evidence suggests that maternal folate and folic acid intake influences pubertal timing in male offspring [46], indicating that folate intervention may help prevent trace metal-related health risks during adolescence [47].

In young adults, only single-metal effects were revealed in Hg, Cd, Se, and Pb. This is consistent with a similar study in U.S. males from NHANES 2013–2016 [21]. Relative to adolescents, who are universally sensitive to metal exposure, we tend to interpret young adults as having a mature endocrine system and a stronger compensatory capacity for heavy metal disruptions. The modifying effects of folate were observed in the association between Pb and 17H in young males. Prior studies found that folate modifies the adverse effects of smoking regarding one-carbon metabolism and redox balance [48], and deficiency in adults can manifest as macrocytic anemia [40]. Folate is still a critical nutrient for adult male health. However, vitamin D did not show a significant modifying effect in adults. The situation was different in a study that concentrated on the serum 25-hydroxyvitamin D (25(OH)D) that mediates the association between heavy metal exposure and cardiovascular disease, and found serum 25(OH)D was negatively associated with Cd, Pb, and Mn, while serum 25(OH)D was positively related to Se [23]. Even though the associations between vitamin D and sex hormones have been identified in EST and TST among males [49], the interactions between vitamin D, heavy metals, and hormones lack high-quality causal evidence. Our findings may provide references for future research.

During aging, the physiological functions of the endocrine system gradually decline, and the secretory patterns of the hypothalamic–pituitary axis change, with alterations in its sensitivity to negative feedback by end hormones [50]. Specifically, HPA axis senescence leads to DHE and AND secretion decrease, and aging in the male HPT axis results in GnRH, TST, AND, and serum inhibin B to FSH ratio reduction, while there are increases in LH and SHBG [50]. Moreover, aging intensifies the deleterious crosstalk between oxidative stress, apoptosis, and inflammation within the gonads, ultimately disrupting sex hormone regulation [51]. Heavy metal exposure may disrupt inflammatory reactions [28] and increase the risk of cognitive impairment in older people [52], all of which may further exacerbate hormonal regulation disorders. As this study found, sex hormones (TST, EST, SHBG, ESO, and ES1) in older adults were disrupted by both mixed and individual heavy metal exposure. Furthermore, vitamin D in older males has not yet exhibited modifying effects, although vitamin D is needed for older adult bone health, its corrected effects may be marked in the phosphorus-, calcium-, and glucose metabolism-related hormones, such as fibroblast growth factor 23, parathyroid, and insulin [53]. In older adults, the modifying effect of folate was found for the association between Se and AND. Folate is a key intermediate of one-carbon metabolism influencing steroid biosynthesis [54] and always plays an essential role across the lifespan.

We have summarized the potential mechanisms linking metal exposure and hormone dysregulation based on a narrative review (Figure 5, Panel A). (1) Epigenetic modifications: Arsenic exposure increases DNA methylation in the promoter region of the steroidogenic factor-1 (SF-1) gene, which significantly suppresses its transcriptional activity and leads to a marked decrease in SF-1 mRNA expression [55,56,57]. SF-1 can regulate the expression of genes such as StAR, CYP19A1, and CYP11A1, thereby influencing steroid hormone synthesis [55]. (2) Hormone-like effects: The properties of the metallic estrogen Cd are similar to those of estradiol, which can induce the transcription and expression of estrogen-related genes [58]. (3) Homocysteine pathway: N-methyl-D-aspartate (NMDA) stimulates the release of FSH and LH [59], while gamma-aminobutyric acid (GABA) plays an important role in regulating the activity of GnRH neurons [60]. As an agonist of NMDA and an antagonist of GABA [61], homocysteine may be elevated by lead exposure, thereby indirectly affecting sex hormone levels [62]. (4) Inhibition of key enzymes: Metal exposure can inhibit the activity of key enzymes and reduce sex hormone synthesis. For example, lead exposure can reduce the production of p450 aromatase messenger RNA and cytochrome p450 aromatase, which is essential for sex hormone formation [63]. (5) Oxidative stress: Metals, such as Cd, can induce the formation of reactive oxygen species and DNA damage, and further reduce testosterone synthesis [64]. (6) Apoptosis or necrosis: Cd exposure can induce apoptosis and necrosis in testicular tissue [65]. Studies also indicate that Pb exposure could induce the expression of caspace-3, an apoptosis-related peptide, eventually increasing the apoptosis proportion in ovarian granulosa cells [66]. (7) Inflammation: Heavy metals, such as Cd, may induce a proinflammatory response [67,68]. Meanwhile, inflammatory mediators such as TNF-α have been reported to play an important role in downregulating SHBG levels [69].

**Figure 6:**
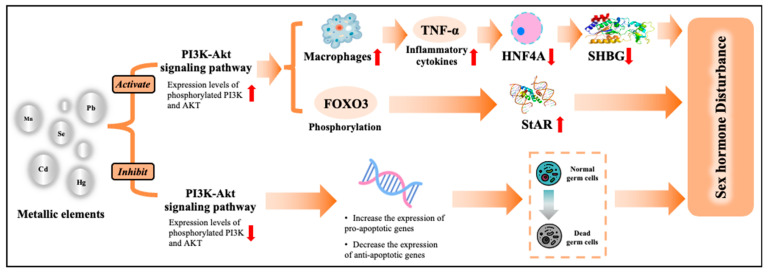
Speculated underlying mechanisms from bioinformatic analysis and narrative review. Abbreviations: Cd, cadmium; Pb, lead; Hg, mercury; Se, selenium; Mn, manganese; PI3K, phosphoinositide 3-kinase; FOXO3, Forkhead box O3; and StAR, steroidogenic acute regulatory.

Furthermore, we conducted bioinformatics analysis to explore the pathways underlying metal exposure and male hormone disturbance, and have identified the *PI3K-Akt* signaling pathway. Metal exposure can induce apoptosis by inactivating the *PI3K-Akt* signaling pathway [70]. A study also reported that metal exposure can activate the *PI3K-Akt* signaling pathway through the expression of miRNA-21, and further induce M1 polarization of macrophages, leading to proinflammatory response [71]. The occurrence of apoptosis or inflammation may further cause sex hormone disturbance [66,69]. The activation of the *PI3K-Akt* signaling pathway can also increase the FOXO3 phosphorylation levels, which, in turn, promote the StAR protein formation and thus affect sex hormone synthesis [72]. Figure 6 summarizes the underlying mechanisms.

The strengths of this study include the use of nationally representative data, which ensures adequate extrapolation to a large population. In addition, the age-stratified analysis allowed us to fully discern the different life-stage responses of hormones affected by metal exposure. Finally, the rigorous results summarized from multiple models strengthen the reliability of our findings. This study also has several limitations. First, the hormone level assay cannot reflect the authentic exposure effects of heavy metals, as the hormones and metal data from the blood were derived at a single time point per participant, while the endocrine disruption by metals always occurs in long-term exposure. Second, given that the cross-sectional study is insufficient for causal inference, this study cannot establish the causal relationship between hormones, metals, and modifiers, which leads us to interpret results with caution. Third, participants with missing values were excluded during data cleaning, which may introduce potential selection bias. Fourth, bioinformatics analysis was not validated in experimental or epidemiological studies. However, results from in silico analysis are useful for hypothesis generation. Finally, limited by sample size in the subgroup analysis, this study explored the effect modification by using the quantile distribution of vitamin D and folate rather than their clinical cutoff value. This methodological choice may reduce the direct clinical applicability of the results.

## 5. Conclusions

This study indicates that exposure to Cd, Pb, Hg, Mn, and Se was predominantly associated with hormonal perturbations (especially TST, EST, SHBG, 17H, AND, ESO, ES1, LH, and PG4) in adolescent and older males. These effects were modified by vitamin D in children and adolescents, whereas by folate across all age groups. The PI3K-Akt signaling pathway was identified as a potential mechanism. Future studies are warranted to reveal the causal relationship between metals and male hormones.

## Figures and Tables

**Table 1:** Characteristic description of the study population.

Variables	All	Subgroups (Years Old)				*p* Value
		[3, 12)	[12, 20)	[20, 50)	[50, 80]	
No.	6547	824	1044	1924	2755	-
Age, years	42 (18, 62)	8 (7, 10)	15 (14, 17)	35 (28, 42)	65 (59, 72)	<0.0001
BMI, kg/m^2^	26.8 (22.6, 31.4)	17.3 (15.7, 20.8)	23.0 (19.7, 27.0)	28.2 (24.8, 33.0)	28.6 (25.4, 32.6)	<0.0001
Education level						0.001
High school or below	1805 (38.58%)	-	-	687 (35.71%)	1118 (40.58%)	
Some college or AA	1414 (30.22%)	-	-	629 (32.69%)	785 (28.49%)	
College graduate or above	1460 (31.20%)	-	-	608 (31.60%)	852 (30.93%)	
Race						<0.0001
Hispanic	1366 (20.86%)	208 (25.24%)	272 (26.05%)	443 (23.02%)	443 (16.08%)	
Non-Hispanic white	3008 (45.94%)	313 (37.99%)	391 (37.45%)	822 (42.72%)	1482 (53.79%)	
Non-Hispanic black	1206 (18.42%)	175 (21.24%)	196 (18.77%)	337 (17.52%)	498 (18.08%)	
Others	967 (14.77%)	128 (15.53%)	185 (17.72%)	322 (16.74%)	332 (12.05%)	
Marital status						<0.0001
Married/with Partner	2937 (62.77%)	-	-	1140 (59.25%)	1797 (65.23%)	
Separated/Never married	1742 (37.23%)	-	-	784 (40.75%)	958 (34.77%)	
Examined time						<0.0001
Morning	3248 (49.61%)	347 (42.11%)	466 (44.64%)	989 (51.40%)	1446 (52.49%)	
Afternoon/evening	3299 (50.39%)	477 (57.89%)	578 (55.36%)	935 (48.60%)	1309 (47.51%)	
Smoke habit						<0.0001
Never	2345 (50.12%)	-	-	1105 (57.43%)	1240 (45.01%)	
Current	1452 (31.03%)	-	-	387 (20.11%)	1065 (38.66%)	
Former	882 (18.85%)	-	-	432 (22.45%)	450 (16.33%)	
Drink habit						<0.0001
Never	286 (6.11%)	-	-	114 (5.93%)	172 (6.24%)	
Current	856 (18.29%)	-	-	198 (10.29%)	658 (23.88%)	
Former	3537 (75.59%)	-	-	1612 (83.78%)	1925 (69.87%)	
PIR	2.5 (1.3, 4.6)	1.8 (0.9, 3.6)	1.8 (0.9, 3.6)	2.8 (1.4, 5.0)	2.8 (1.5, 5.0)	<0.0001
Cadmium, μg/L	0.18 (0.11, 0.34)	0.08 (0.07, 0.12)	0.13 (0.08, 0.17)	0.18 (0.12, 0.35)	0.28 (0.18, 0.49)	<0.0001
Lead, μg/dL	0.75 (0.46, 1.26)	0.46 (0.33, 0.65)	0.42 (0.31, 0.58)	0.69 (0.47, 1.06)	1.13 (0.77, 1.71)	<0.0001
Mercury, μg/L	0.47 (0.20, 1.13)	0.20 (0.18, 0.37)	0.21 (0.20, 0.54)	0.56 (0.22, 1.28)	0.70 (0.31, 1.55)	<0.0001
Manganese, μg/L	8.73 (7.10, 10.82)	9.78 (8.01, 12.08)	9.65 (7.93, 11.65)	8.47 (6.96, 10.36)	8.31 (6.71, 10.34)	<0.0001
Selenium, μg/L	180.25 (165.75, 196.59)	165.28 (154.1, 178.87)	179.34 (166.78, 193.32)	184.55 (170.95, 199.70)	182.08 (167.2, 198.87)	<0.0001

-: Not available.

**Table 2:** Findings summary from the three models to reveal individual metal exposure and hormones.

Sex Hormones	Children	Adolescents	Young Adults	Older Adults
TST		Cd (+)	Hg (+)	Cd (+)
EST		Cd (+); Se (~)		Cd (+); Mn (+)
SHBG		Mn (−); Se (−)	Cd (+); Se (−)	Cd(~); Pb(~); Se(~)
17H		Se (+)		
AND		Cd (+)		Mn (+)
AMH				
ESO	Pb (−)	Cd (+)		Cd(+); Pb(+); Mn(+); Se (−)
ES1		Pb (~)	Se (+)	
FSH	Mn (+)			Hg (~)
LH		Cd (+)	Pb (+)	
PG4		Se (+); Hg (+)	Pb (+)	
DHE		Se (+)		Hg (+)

Abbreviations: TST, testosterone; EST, estradiol; SHBG, sex hormone-binding globulin; 17H, 17α-hydroxyprogesterone; AND, androstenedione; AMH, anti-Müllerian hormone; ESO, estrone; ES1, estrone sulfate; FSH, follicle-stimulating hormone; LH, luteinizing hormone; PG4, progesterone; DHE, dehydroepiandrosterone sulfate; Cd, cadmium; Pb, lead; Hg, mercury; Se, selenium; Mn, manganese. Note: “+” indicates the association was positively significant; “−” indicates the association was negatively significant; “~” indicates the association was nonlinearly significant. Bold font indicates the results were consistent across all three models.

## Data Availability

The data are available through the following link: https://wwwn.cdc.gov/nchs/nhanes/default.aspx (accessed on 24 July 2025).

## Supplementary Materials

The following supporting information can be downloaded at: https://www.mdpi.com/article/10.3390/toxics14010055/s1, Table S1. Results summary for individual exposure effect among children. Table S2. Results summary for individual exposure effect among adolescents. Table S3. Results summary for individual exposure effect among young adults. Table S4. Results summary for individual exposure effect among older adults. Figure S1. Flow chart of this study participants inclusion process. Figure S2. Flow chart of the bioinformatic analysis. Figure S3. Correlation between sex hormones and metals. Figure S4. The distribution of sex hormones across different age groups. Figure S5. Exposure-response relationship between metal exposure and sex hormones in participants aged between 3 and 11 years old. Figure S6. Exposure-response relationship between metal exposure and sex hormones in participants aged between 12 and 19 years old. Figure S7. Exposure-response relationship between metal exposure and sex hormones in participants aged between 20 and 49 years old. Figure S8. Exposure-response relationship between metal exposure and sex hormones in participants aged between 50 and 80 years old. Figure S9. Effect modification of folate in the association between metals and sex hormones. Figure S10. Sensitive analysis: heatmap for the effect of metal exposure on sex hormones across different age groups. Figure S11. Discrimination ability of metal-associated hormones to a certain metal exposure.
