# Supplementary material for: Age-Dependent Effects of Heavy Metals on the Hypothalamic–Pituitary–Testicular Axis-Related Hormones in Men
Source: Toxics. 2026 Jan 7;14(1):55. doi: 10.3390/toxics14010055 (PMC12846245; doi:10.3390/toxics14010055)
Supplement: Supplementary file 1 [file toxics-14-00055-s001.zip › toxics-3973694-supplementary.pdf]

Supplementary file

Tables

Table S1. Results summary for individual exposure effect among children.
Table S2. Results summary for individual exposure effect among adolescents.
Table S3. Results summary for individual exposure effect among young adults.
Table S4. Results summary for individual exposure effect among older adults.

Figures

Figure S1. Flow chart of this study participants inclusion process.
Figure S2. Flow chart of the bioinformatic analysis.
Figure S3. Correlation between sex hormones and metals.
Figure S4. The distribution of sex hormones across different age groups.
Figure S5. Exposure-response relationship between metal exposure and sex hormones in participants aged between 3 and 11 years old.
Figure S6. Exposure-response relationship between metal exposure and sex hormones in participants aged between 12 and 19 years old.
Figure S7. Exposure-response relationship between metal exposure and sex hormones in participants aged between 20 and 49 years old.
Figure S8. Exposure-response relationship between metal exposure and sex hormones in participants aged between 50 and 80 years old.
Figure S9. Effect modification of folate in the association between metals and sex hormones.
Figure S10. Sensitive analysis: heatmap for the effect of metal exposure on sex hormones across different age groups.
Figure S11. Discrimination ability of metal-associated hormones to a certain metal exposure.

Table S1. Results summary for individual exposure effect among children.

Sex hormones	Multiple linear regression		RCS	Summary
	Continuous	Categorical		
TST		Mn(+)		
EST		Pb(+)	Cd(+)	
SHBG				
17H				
AND		Se(+)		
AMH		Hg(+)		ESO-Pb(-); FSH-Mn(+)
ESO	Pb (-)	Pb(-)		
ES1		Mn(-);Se(+)		
FSH	Mn (+)	Mn(+)	Cd(~)	
LH		Mn(+);Hg(+)		
PG4				
DHE				

Abbreviations: TST, testosterone; EST, estradiol; SHBG, Sex hormone-binding globulin; 17H, 17α-hydroxyprogesterone; AND, androstenedione; AMH, anti-Müllerian hormone; ESO, estrone; ES1, estrone sulfate; FSH, follicle-stimulating hormone; LH, luteinizing hormone; PG4, progesterone; DHE, dehydroepiandrosterone sulfate; Cd, Cadmium; Pb, Lead; Hg, Mercury; Se, Selenium; Mn, Manganese; RCS, Restricted cubic splines.

Note: "+" indicate the association was positively significant; "-" indicate the association was negatively significant; "~" indicate the association was nonlinearly significant.

Table S2. Results summary for individual exposure effect among adolescents.

Sex hormones	Multiple linear regression		RCS	Summary
	Continuous	Categorical		
TST	Cd (+)	Cd(+);Mn(+);Hg(-)	Pb(~);Cd (+)	TST-Cd(+); EST-Cd(+); EST-Se(~); SHBG-Mn(-); SHBG-Se(-); 17H-Se(+); AND-Cd(+); ESO-Cd(+); ES1-Pb(~); LH-Cd(+); PG4-Se(+); PG4-Hg(+); DHE-Se(+)
EST	Cd(+);Se(+)	Cd (+)	Cd (+);Se(~)	
SHBG	Mn(-);Se(-)	Pb(+);Mn(-);Se(-)	Hg(~);Se(-);Mn(-)	
17H	Cd(+);Se(+)	Se(+)	Se(+)	
AND	Cd (+)	Cd (+)	Cd (+);Se(+)	
AMH		Cd (-)		
ESO	Cd (+)	Cd (+)	Pb(~);Cd (+);Se(~)	
ES1		Pb(-)	Pb(~);Se(+)	
FSH		Cd (+)	Pb(+)	
LH	Cd (+)	Cd (+);Pb(+)	Cd (+);Hg(~);Se(+)	
PG4	Hg(+);Se(+)	Se(+)	Hg(+);Se(+)	
DHE	Se(+)	Se(+)		

Abbreviations: TST, testosterone; EST, estradiol; SHBG, Sex hormone-binding globulin; 17H, 17α-hydroxyprogesterone; AND, androstenedione; AMH, anti-Müllerian hormone; ESO, estrone; ES1, estrone sulfate; FSH, follicle-stimulating hormone; LH, luteinizing hormone; PG4, progesterone; DHE, dehydroepiandrosterone sulfate; Cd, Cadmium; Pb, Lead; Hg, Mercury; Se, Selenium; Mn, Manganese; RCS, Restricted cubic splines.

Note: "+" indicate the association was positively significant; "-" indicate the association was negatively significant; "~" indicate the association was nonlinearly significant.

Table S3. Results summary for individual exposure effect among young adults.

Sex hormones	Multiple linear regression		RCS	Summary
	Continuous	Categorical		
TST		Cd(+);Mn(-);Hg(+)	Pb(~);Hg(+)	TST-Hg(+); SHBG-Cd(+); SHBG-Se(-); ES1-Se(+); LH-Pb(+); PG4-Pb(+)
EST		Mn(-)	Hg(+)	
SHBG	Cd(+);Se(-)	Cd(+);Se(-)	Cd(+);Se(-);Mn(~)	
17H		Pb(+)		
AND		Pb(+)	Cd(+)	
AMH			Se(~)	
ESO				
ES1		Pb(+);Se(+)	Se(+)	
FSH				
LH		Pb(+)	Pb(+);Se(~)	
PG4	Pb(+)	Pb(+)	Pb(+)	
DHE		Cd(+)		

Abbreviations: TST, testosterone; EST, estradiol; SHBG, Sex hormone-binding globulin; 17H, 17α-hydroxyprogesterone; AND, androstenedione; AMH, anti-Müllerian hormone; ESO, estrone; ES1, estrone sulfate; FSH, follicle-stimulating hormone; LH, luteinizing hormone; PG4, progesterone; DHE, dehydroepiandrosterone sulfate; Cd, Cadmium; Pb, Lead; Hg, Mercury; Se, Selenium; Mn, Manganese; RCS, Restricted cubic splines.

Note: "+" indicate the association was positively significant; "-" indicate the association was negatively significant; "~" indicate the association was nonlinearly significant.

Table S4. Results summary for individual exposure effect among older adults.

Sex hormones	Multiple linear regression		RCS	Summary
	Continuous	Categorical		
TST		Cd(+);Se(-)	Cd(+)	TST-Cd(+);<br>EST-Cd(+);<br>EST-Mn(+);<br>SHBG-Cd(~);<br>SHBG-Pb(~);<br>SHBG-Se(~);<br>AND-Mn(+);<br>ESO-Cd(+);<br>ESO-Pb(+);<br>ESO-Mn(+);<br>ESO-Se(-);<br>FSH-Hg(~);<br>DHE-Hg(+)
EST	Cd(+);Mn(+)	Cd(+);Mn(+);Se(-)	Pb(+);Cd(+);Mn(+)	
SHBG	Cd(+);Pb(+);Se(-)	Cd(+);Pb(+);Se(-)	Pb(~);Cd(~);Se(~);Mn(~)	
17H				
AND		Pb(+);Mn(+)	Se(-);Mn(+)	
AMH		Cd(+);Pb(+)		
ESO	Cd(+);Pb(+);Mn(+);Se(-)	Pb(+);Mn(+);Hg(+);Se(-)	Pb(+);Cd(+);Se(-);Mn(+)	
ES1			Pb(~);Se(+)	
FSH		Hg(-)	Hg(~)	
LH			Cd(+)	
PG4			Hg(-)	
DHE		Cd(+);Pb(+);Hg(+)	Hg(+);Mn(~)	

Abbreviations: TST, testosterone; EST, estradiol; SHBG, Sex hormone-binding globulin; 17H, 17α-hydroxyprogesterone; AND, androstenedione; AMH, anti-Müllerian hormone; ESO, estrone; ES1, estrone sulfate; FSH, follicle-stimulating hormone; LH, luteinizing hormone; PG4, progesterone; DHE, dehydroepiandrosterone sulfate; Cd, Cadmium; Pb, Lead; Hg, Mercury; Se, Selenium; Mn, Manganese; RCS, Restricted cubic splines.

Note: "+" indicate the association was positively significant; "-" indicate the association was negatively significant; "~" indicate the association was nonlinearly significant.

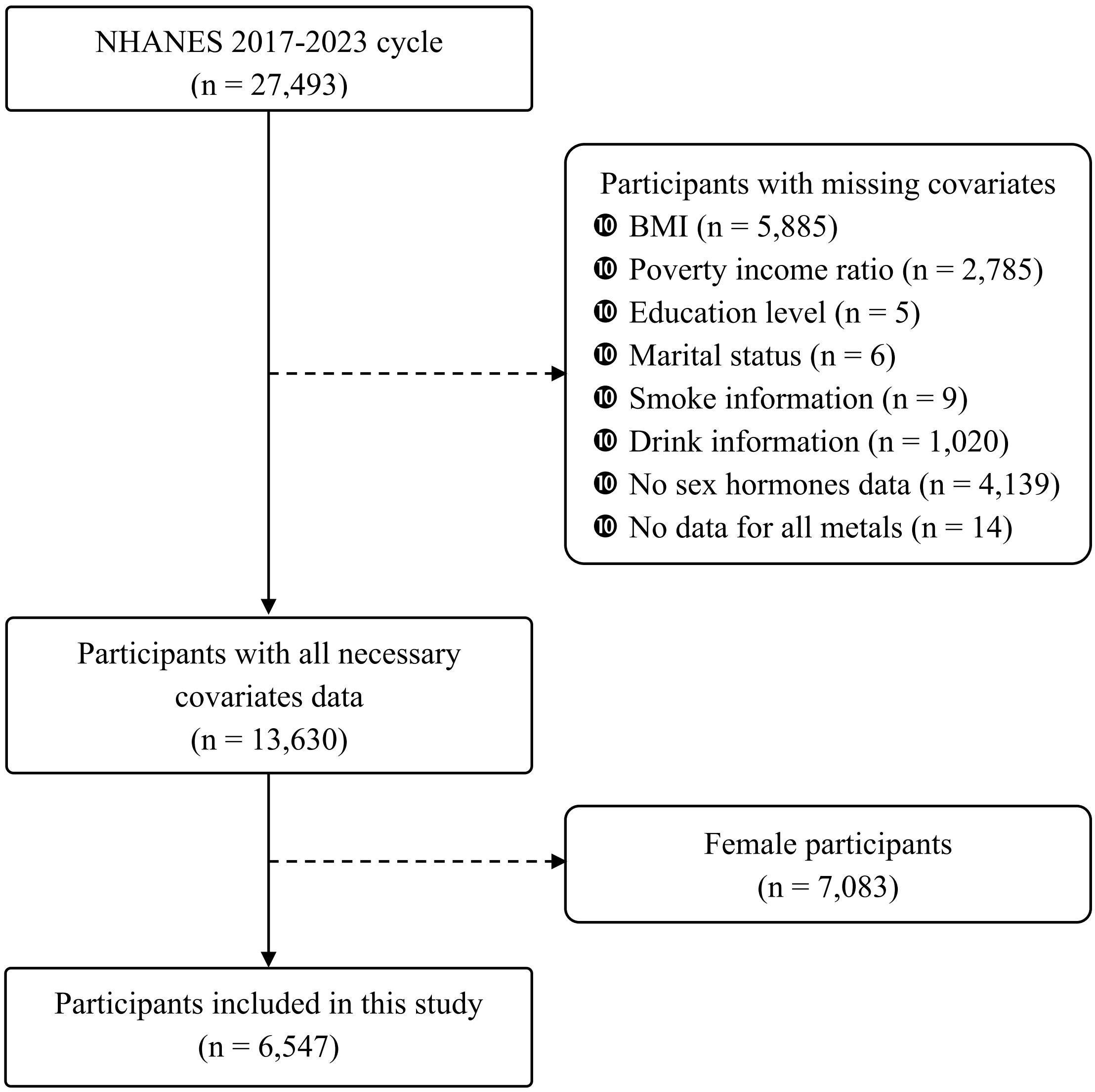

Figure S1. Flow chart of this study participants inclusion process.

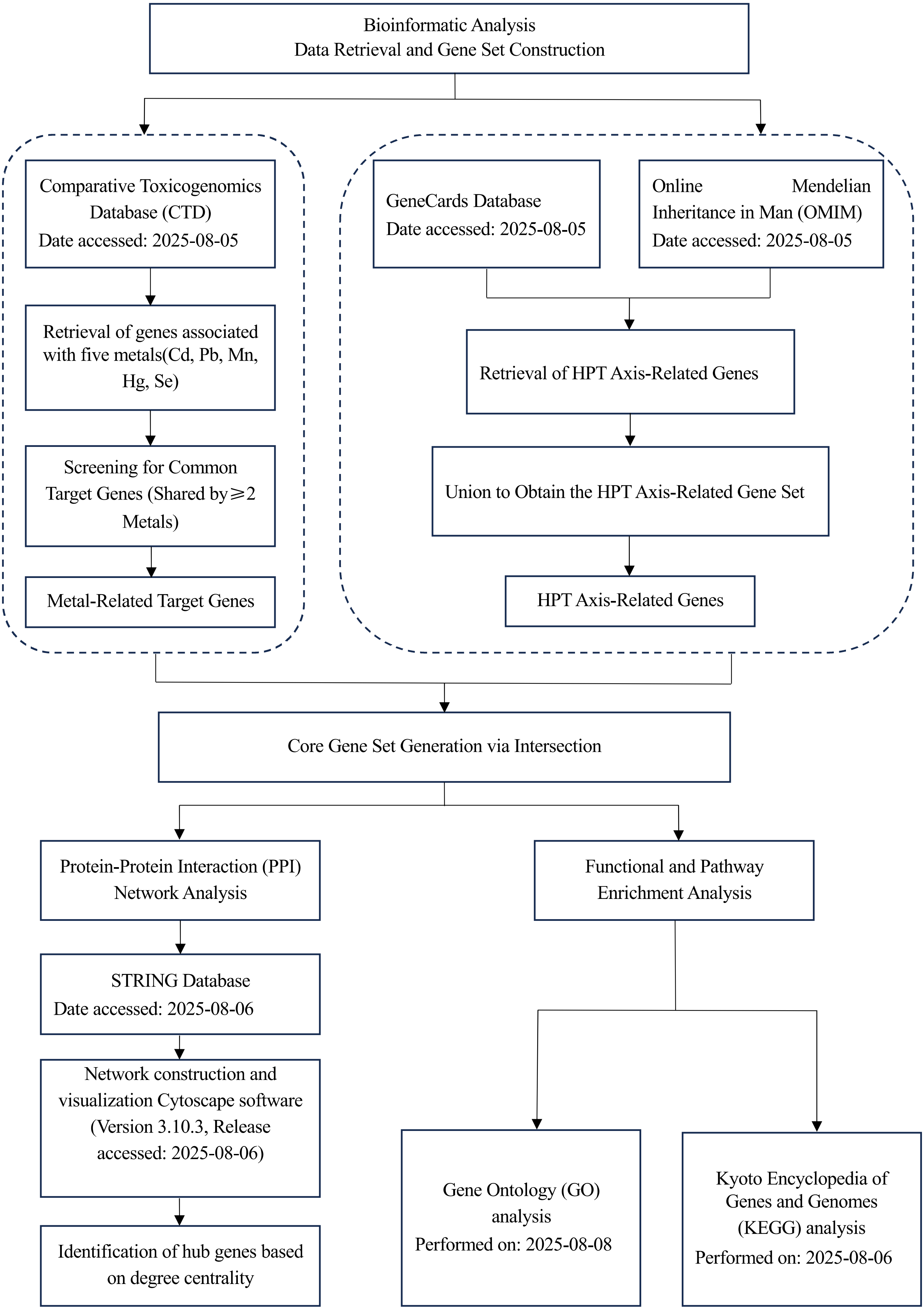

Figure S2. Flow chart of the bioinformatic analysis.

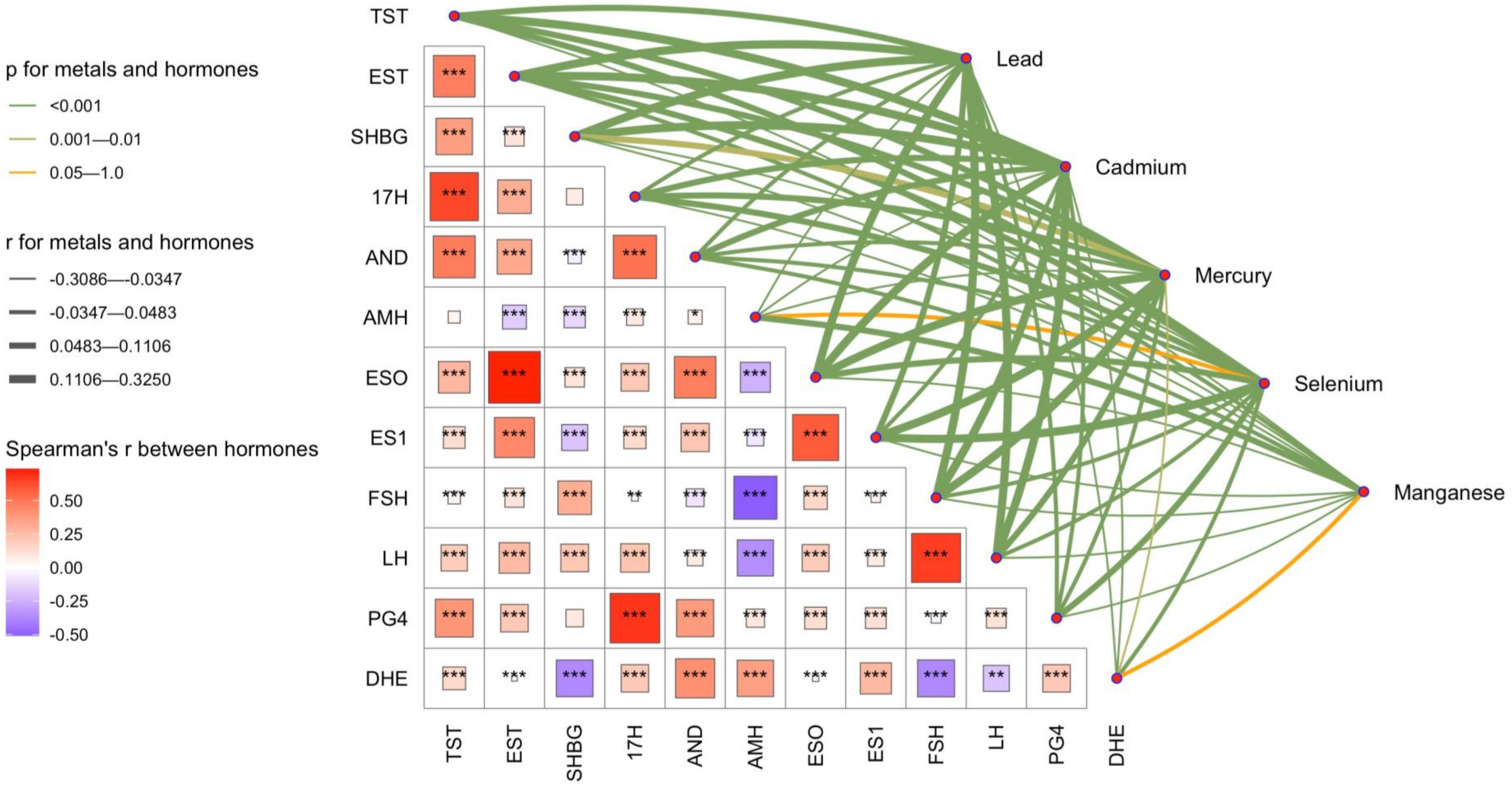

Figure S3. Correlation between sex hormones and metals.

Abbreviations: TST, testosterone; EST, estradiol; SHBG, Sex hormone-binding globulin; 17H, 17α-hydroxyprogesterone; AND, androstenedione; AMH, anti-Müllerian hormone; ESO, estrone; ES1, estrone sulfate; FSH, follicle-stimulating hormone; LH, luteinizing hormone; PG4, progesterone; DHE, dehydroepiandrosterone sulfate. Note: * indicates the *P* value < 0.05; ** indicates the *P* value < 0.005; *** indicates the *P* value < 0.0001.

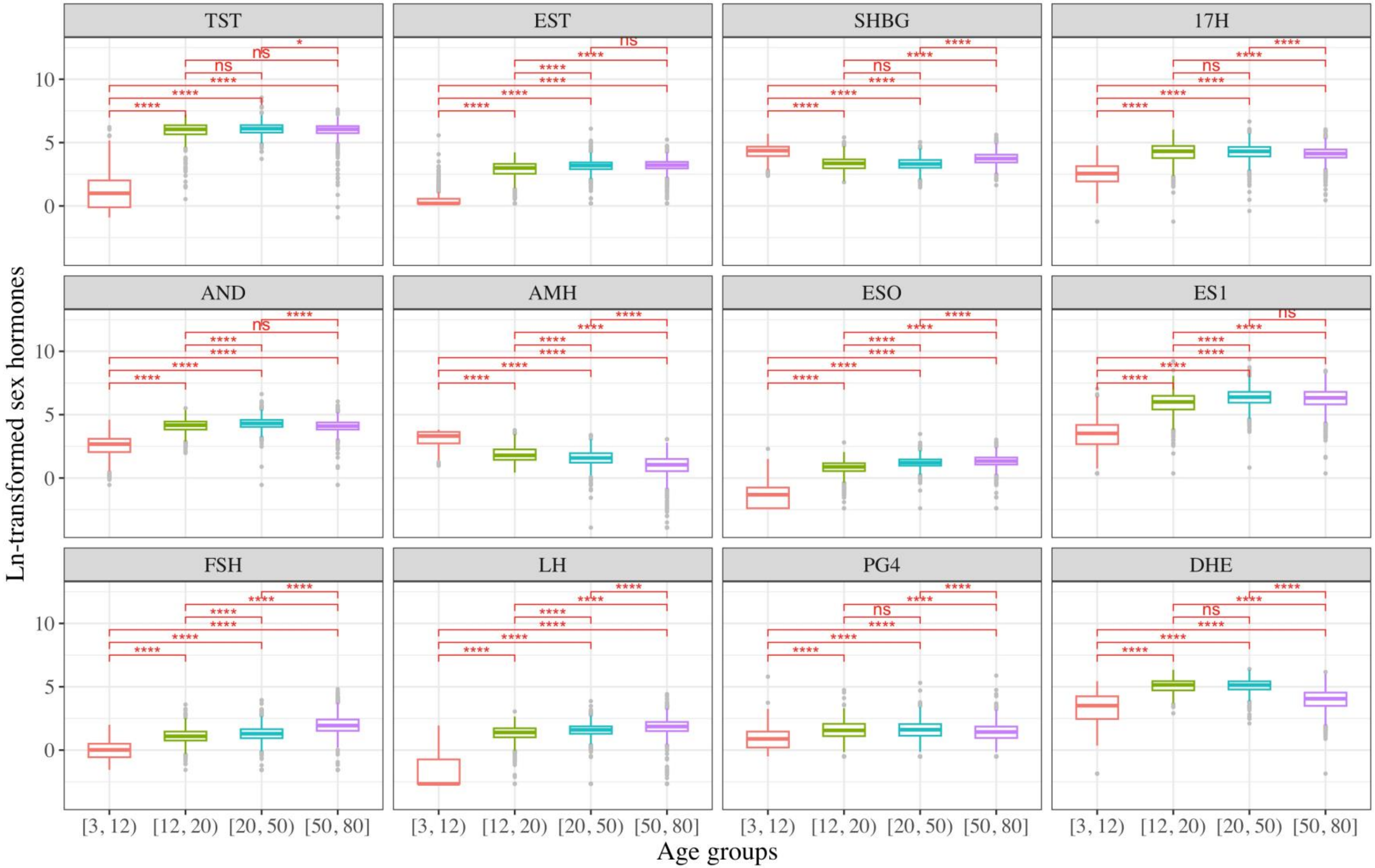

Figure S4. The distribution of sex hormones across different age groups.

Abbreviations: TST, testosterone; EST, estradiol; SHBG, Sex hormone-binding globulin; 17H, 17α-hydroxyprogesterone; AND, androstenedione; AMH, anti-Müllerian hormone; ESO, estrone; ES1, estrone sulfate; FSH, follicle-stimulating hormone; LH, luteinizing hormone; PG4, progesterone; DHE, dehydroepiandrosterone sulfate.

Note: * indicates the *P* value < 0.05; **** indicates the *P* value < 0.0001.

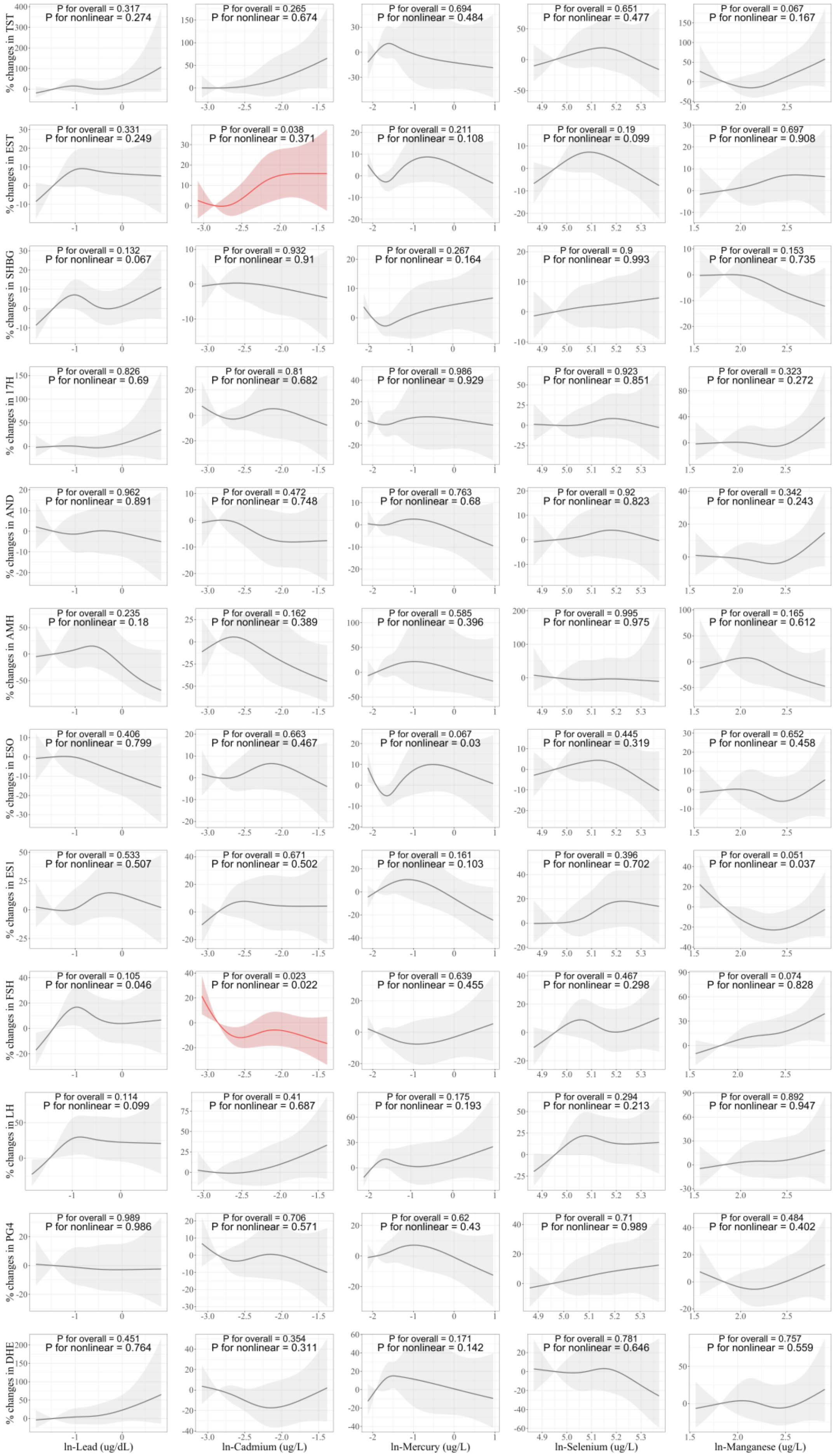

Figure S5. Exposure-response relationship between metal exposure and sex hormones in participants aged between 3 and 11 years old

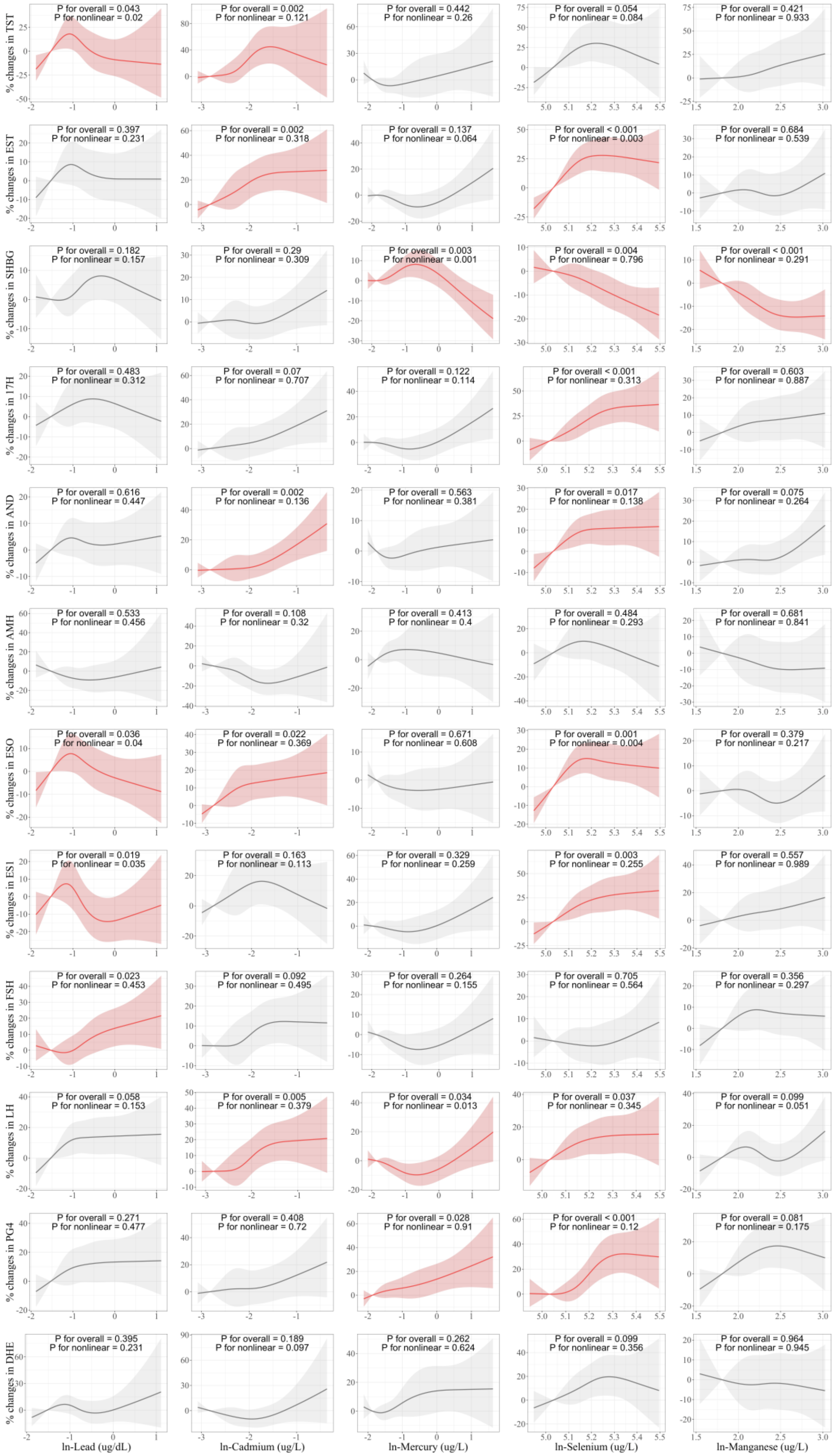

Figure S6. Exposure-response relationship between metal exposure and sex hormones in participants aged between 12 and 19 years old

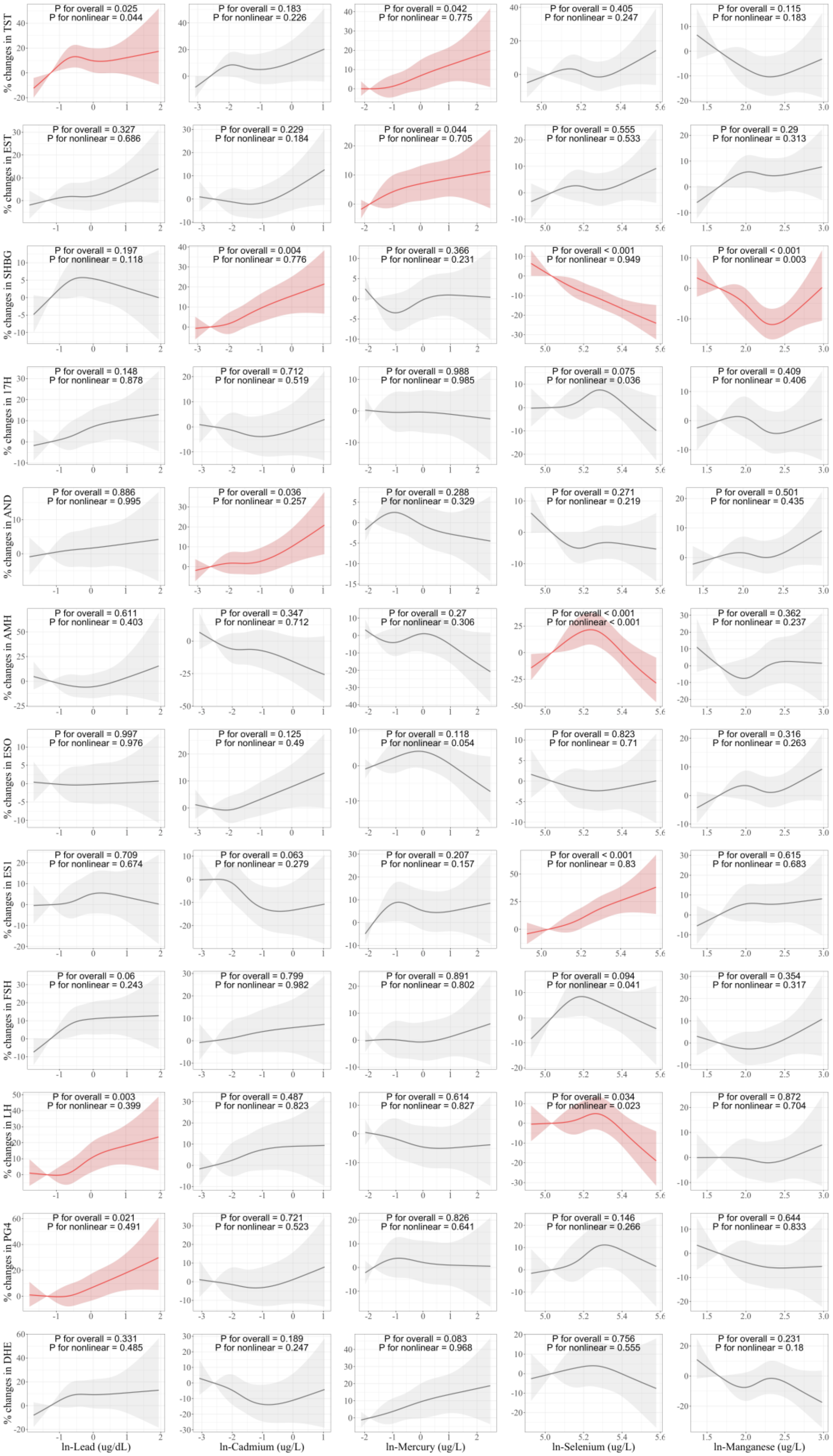

Figure S7. Exposure-response relationship between metal exposure and sex hormones in participants aged between 20 and 49 years old

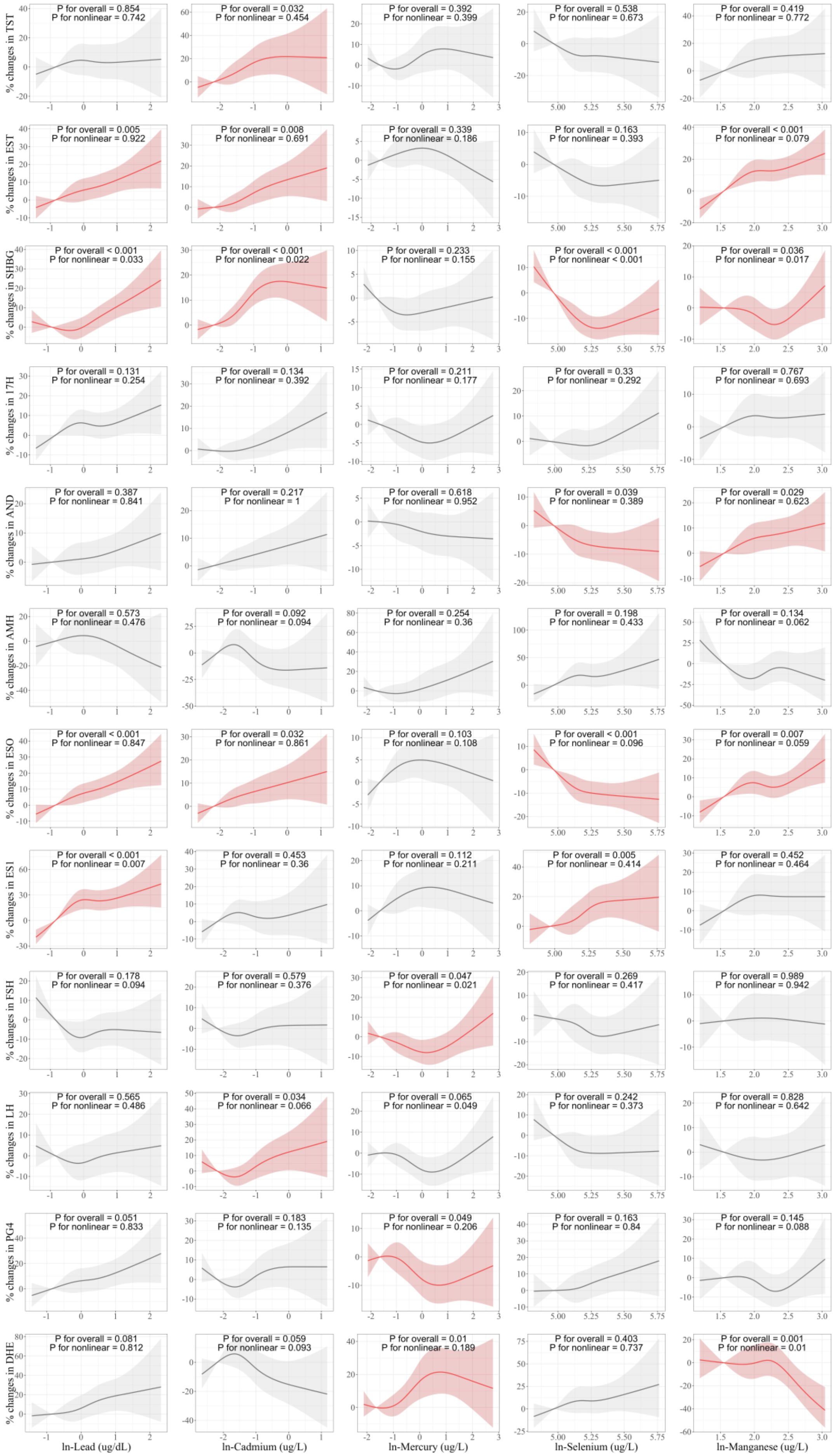

Figure S8. Exposure-response relationship between metal exposure and sex hormones in participants aged between 50 and 80 years old

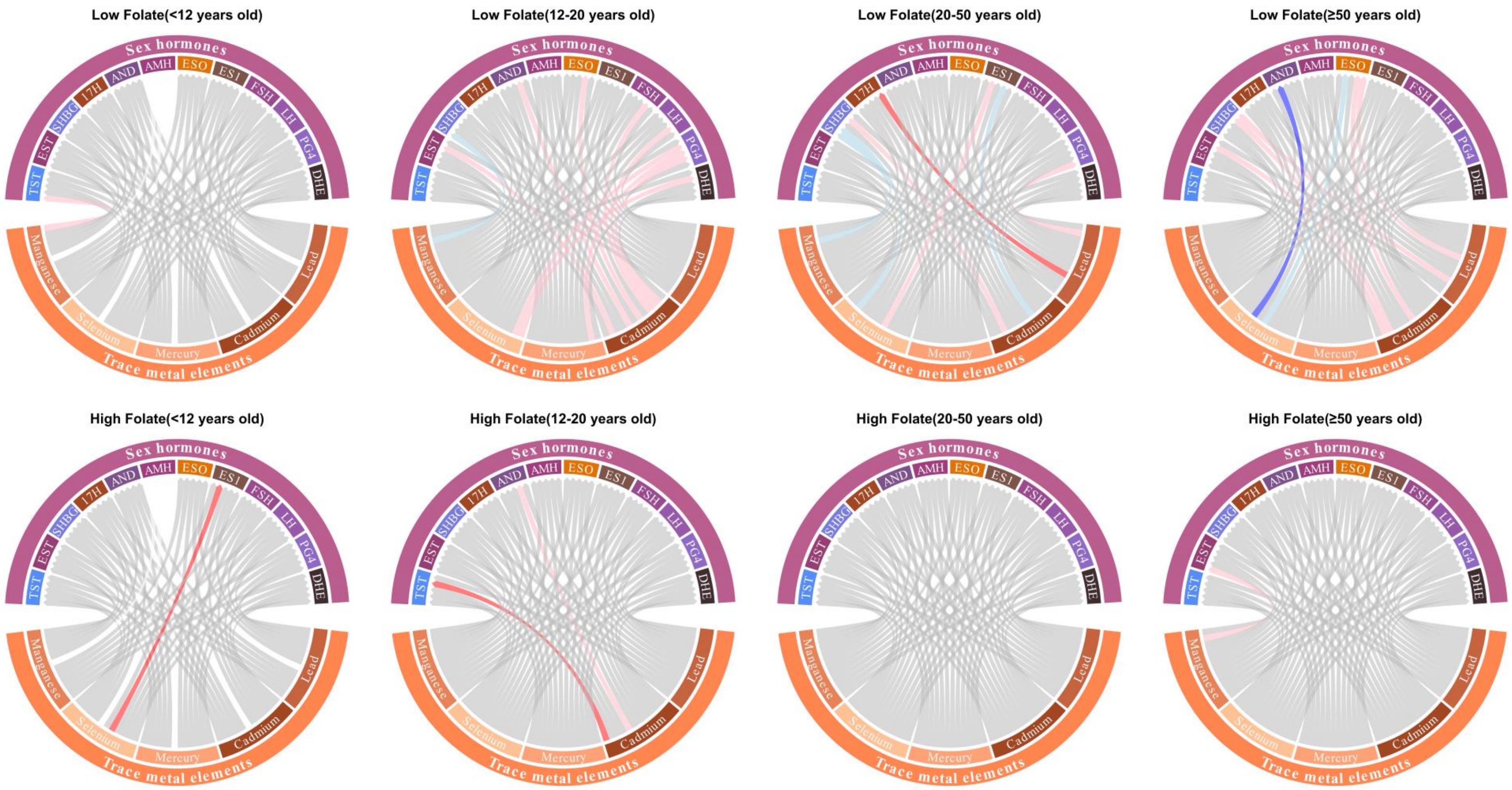

Figure S9. Effect modification of folate in the association between metals and sex hormones

Abbreviations: TST, testosterone; EST, estradiol; SHBG, Sex hormone-binding globulin; 17H, 17α-hydroxyprogesterone; AND, androstenedione; AMH, anti-Müllerian hormone; ESO, estrone; ES1, estrone sulfate; FSH, follicle-stimulating hormone; LH, luteinizing hormone; PG4, progesterone; DHE, dehydroepiandrosterone sulfate. Note: The red line indicates a significant positive association between the corresponding metal-hormone pair, and a significant difference in effect estimates when compared to the same pair in the counterpart group. The pink line, by contrast, denotes a significant positive association for the pair but no significant difference in effect estimates relative to the same pair in the counterpart group. The dark blue line signifies a significant negative association between the corresponding metal-hormone pair, and a significant difference in effect estimates when compared to the same pair in the counterpart group. Conversely, the light blue line indicates a significant negative association for the pair but no significant difference in effect estimates relative to the same pair in the counterpart group.

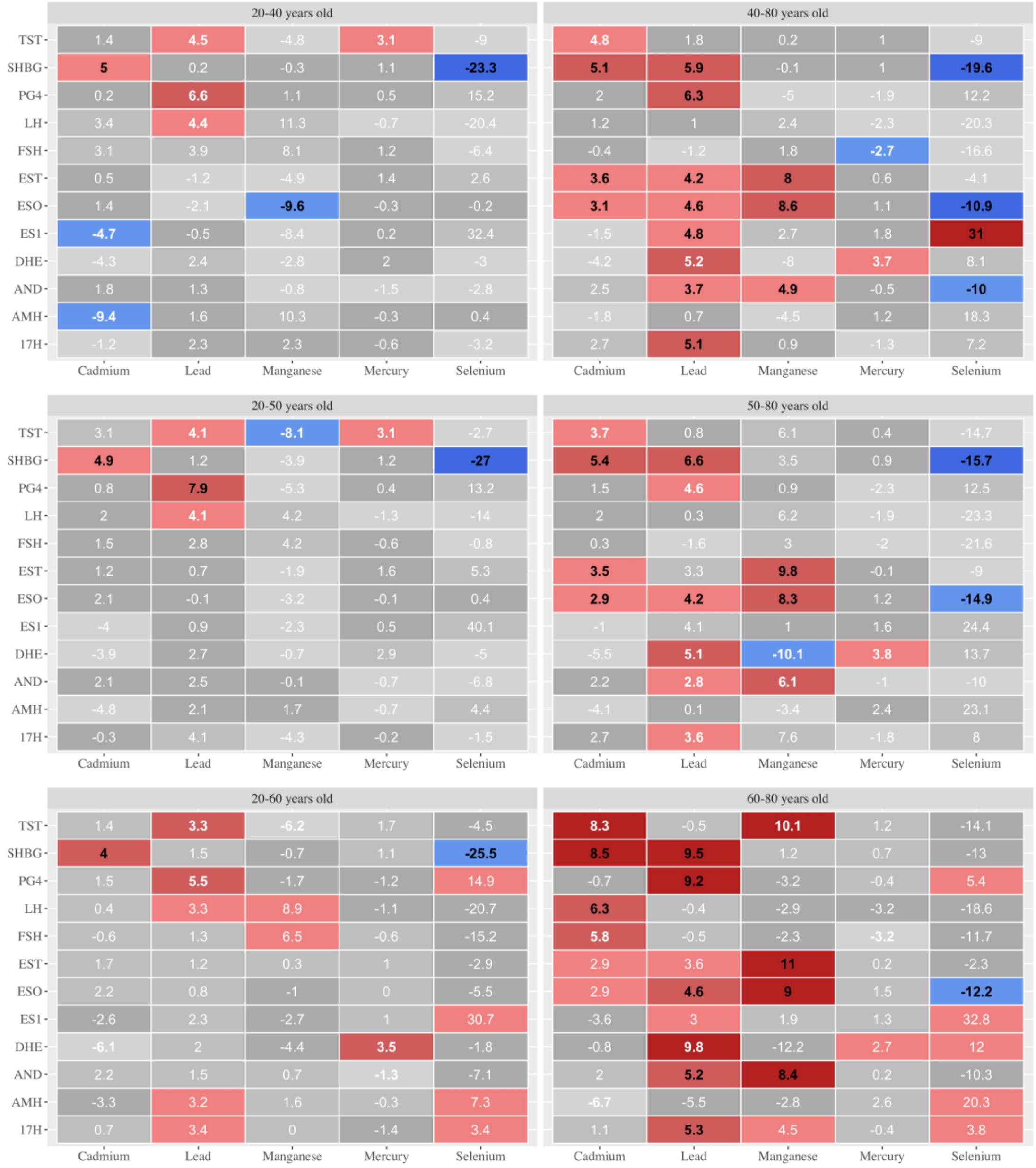

Figure S10. Sensitive analysis: heatmap for the effect of metal exposure on sex hormones across different age groups.

Abbreviations: TST, testosterone; EST, estradiol; SHBG, Sex hormone-binding globulin; 17H, 17α-hydroxyprogesterone; AND, androstenedione; AMH, anti-Müllerian hormone; ESO, estrone; ES1, estrone sulfate; FSH, follicle-stimulating hormone; LH, luteinizing hormone; PG4, progesterone; DHE, dehydroepiandrosterone sulfate.

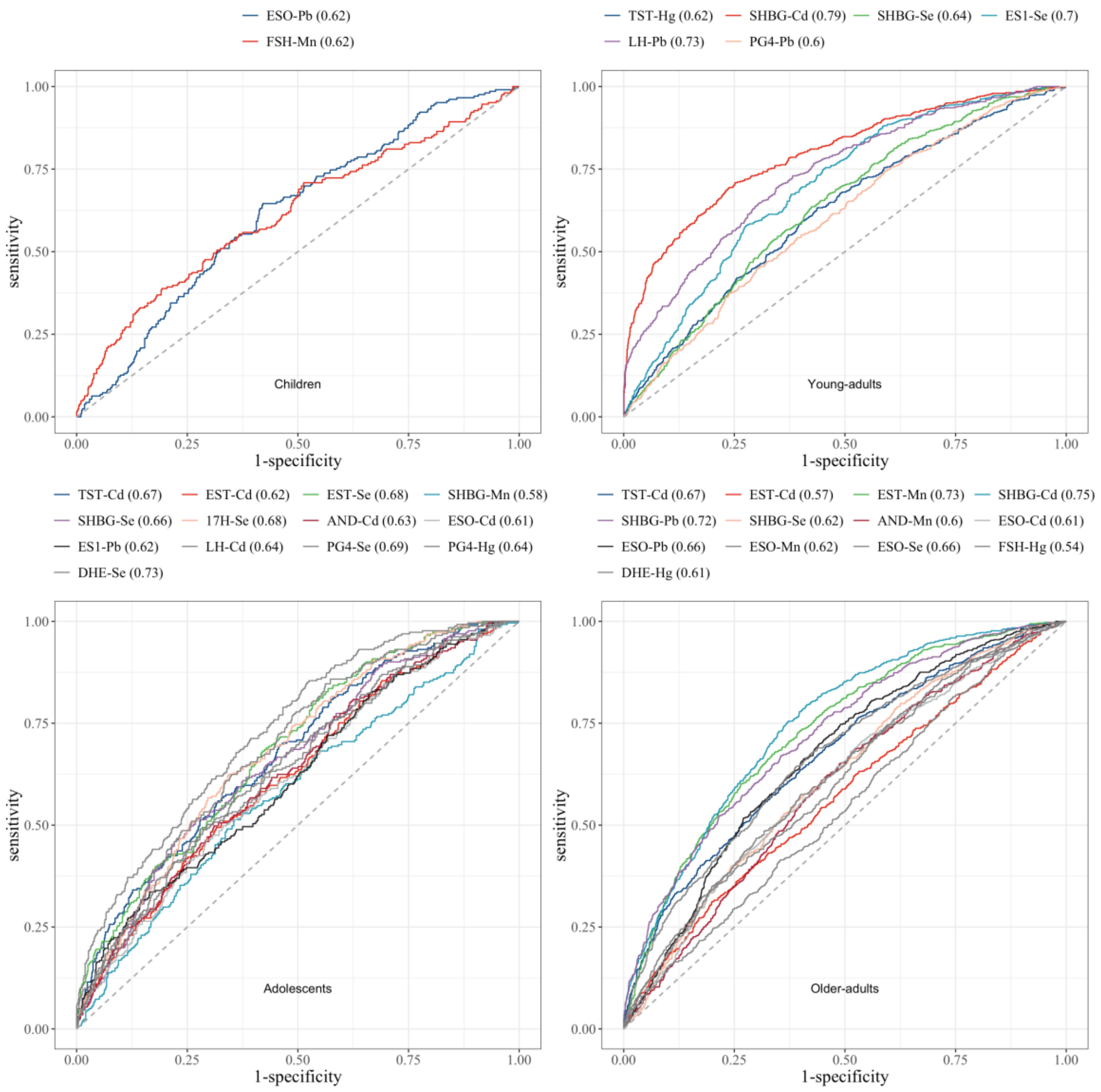

Figure S11. Discrimination ability of metal-associated hormones to a certain metal exposure

Abbreviations: TST, testosterone; EST, estradiol; SHBG, Sex hormone-binding globulin; 17H, 17α-hydroxyprogesterone; AND, androstenedione; AMH, anti-Müllerian hormone; ESO, estrone; ES1, estrone sulfate; FSH, follicle-stimulating hormone; LH, luteinizing hormone; PG4, progesterone; DHE, dehydroepiandrosterone sulfate; Cd, Cadmium; Pb, Lead; Hg, Mercury; Se, Selenium; Mn, Manganese
